# Unique expression and critical role of metallothionein 3 in the control of osteoclastogenesis and osteoporosis

**DOI:** 10.1038/s12276-024-01290-3

**Published:** 2024-08-01

**Authors:** Shenzheng Mo, Min Kyung Kim, Ji Sun Jang, Seung Hye Lee, Seo Jin Hong, Suhan Jung, Hong-Hee Kim

**Affiliations:** 1 Department of Cell and Developmental Biology, School of Dentistry, Seoul National University, Seoul, 03080 Republic of Korea; 2 Dental Research Institute, School of Dentistry, Seoul National University, Seoul, 03080 Republic of Korea; 3 Bone Science R&D Center, Tissue Regeneration Institute, Osstem Implant, Seoul, 07789 Republic of Korea

**Keywords:** Targeted bone remodelling, Transcriptomics

## Abstract

Bone homeostasis is maintained by an intricate balance between osteoclasts and osteoblasts, which becomes disturbed in osteoporosis. Metallothioneins (MTs) are major contributors in cellular zinc regulation. However, the role of MTs in bone cell regulation has remained unexplored. Single-cell RNA sequencing analysis discovered that, unlike the expression of other MT members, the expression of MT3 was unique to osteoclasts among various macrophage populations and was highly upregulated during osteoclast differentiation. This unique MT3 upregulation was validated experimentally and supported by ATAC sequencing data analyses. Downregulation of MT3 by gene knockdown or knockout resulted in excessive osteoclastogenesis and exacerbated bone loss in ovariectomy-induced osteoporosis. Transcriptome sequencing of MT3 knockdown osteoclasts and gene set enrichment analysis indicated that the oxidative stress and redox pathways were enriched, which was verified by MT3-dependent regulation of reactive oxygen species (ROS). In addition, MT3 deficiency increased the transcriptional activity of SP1 in a manner dependent on intracellular zinc levels. This MT3-zinc-SP1 axis was crucial for the control of osteoclasts, as zinc chelation and SP1 knockdown abrogated the promotion of SP1 activity and osteoclastogenesis by MT3 deletion. Moreover, SP1 bound to the NFATc1 promoter, and overexpression of an inactive SP1 mutant negated the effects of MT3 deletion on NFATc1 and osteoclastogenesis. In conclusion, MT3 plays a pivotal role in controlling osteoclastogenesis and bone metabolism via dual axes involving ROS and SP1. The present study demonstrated that MT3 elevation is a potential therapeutic strategy for osteolytic bone disorders, and it established for the first time that MT3 is a crucial bone mass regulator.

## Zinc Regulator MT3: Implications for Osteoporosis Treatment

Bone diseases such as osteoporosis often result from imbalances in bone remodeling, a process involving bone breakdown by cells called osteoclasts and formation by cells called osteoblasts. This study examines the role of Metallothionein 3, a protein that binds to zinc, in osteoclasts. Using a mix of single-cell RNA sequencing database and knockout mouse models, the study investigates how MT3 affects osteoclast development and activity. The researchers used various methods, including gene knockdown and overexpression techniques, to alter MT3 levels in cells and observed the effects on osteoclast formation and bone breakdown. The results indicate that MT3 inhibits osteoclast development and decreases bone loss, suggesting its potential as a treatment target for bone diseases. The study concludes that MT3 plays a crucial role in bone remodeling by controlling osteoclast activity.

This summary was initially drafted using artificial intelligence, then revised and fact-checked by the author.

## Introduction

Bone is a tissue that undergoes remodeling through bone degradation by osteoclasts and new bone synthesis by osteoblasts^1,2^. Disturbances in the regulatory network of bone remodeling cause various bone diseases, including osteoporosis. Osteoporosis, which is prevalent among postmenopausal women and elderly individuals, markedly increases the risk of fractures and substantially burdens health care systems and society^3,4^. Despite the recent development of some therapeutic agents for osteoporosis, limitations in their use due to side effects and poor compliance have increased the necessity for alternative therapies based on diverse mechanisms of action^5,6^. Therefore, elucidating new targets through a deeper understanding of the molecular and cellular characteristics of bone cell generation is needed.

Osteoclasts are specialized macrophages that function in hard tissue catabolism for bone remodeling and blood calcium supply^2^. These bone-resorptive cells are generated from monocyte lineage progenitors through differentiation driven by receptor activators of nuclear factor-kappa B ligand (RANKL)^7^. The differentiation process comprises a commitment to osteoclast precursors, proliferation of the precursors, cell-to-cell fusion, and polarization to active cells^8^. The signals from RANK, the receptor for RANKL, and the macrophage colony-stimulating factor (M-CSF) receptor are transduced to multiple intracellular targets, resulting in the activation of the MAPK, IKK-NFκB, and Ca^2+^-calcineurin pathways^7,8^. These signaling pathways lead to the induction and stimulation of the c-Fos, NFκΒ, and NFATc1 transcription factors, which are essential for osteoclastogenesis^7–10^. NFATc1, together with other transcription factors, upregulates the expression of osteoclast marker genes, including *Acp5* (encodes tartrate-resistant acid phosphatase, TRAP), *Ctsk* (cathepsin K), and *Mmp9* (matrix metalloproteinase-9)^7–10^. RANKL signaling also evokes the generation of reactive oxygen species (ROS) by activating NADPH oxidases^11^ and increasing mitochondrial biogenesis^12^. As a second messenger, ROS contribute to the activation of MAPKs and NFκB by RANKL^11,13^.

Bone, as an important reservoir for various metal ions, contains approximately 30% of the total zinc in the human body^14–16^. Although the humoral factors that regulate systemic zinc homeostasis are elusive, more than 30 proteins have been identified to regulate cellular zinc homeostasis^14–16^. Cellular zinc, as a structural component and catalytic cofactor of numerous proteins, including transcription factors, plays crucial roles in diverse aspects of cell responses^16,17^. In addition, zinc acts as a first or second messenger in the nervous and immune systems^18,19^. Intracellular free zinc (Zn^2+^) levels are largely regulated by metallothioneins (MTs) and zinc transporters^15,20^. MTs are a family of small cysteine-rich proteins that bind heavy metals and provide protection against oxidative stress^21–23^. MTs form Zn-MT complexes by binding up to seven Zn^2+^ ions, with three bound to the N-terminal β-domain and four bound to the C-terminal α-domain^21^. The MT family encompasses four members, namely, MT1, MT2, MT3, and MT4. MT1 and MT2 are ubiquitously expressed, whereas MT3 is found primarily in the brain^21,24,25^. In addition to its unique distribution and structure, MT3 does not typically respond to metal ions or other common inducers^24–27^. Moreover, MT3 has a stronger scavenging effect on hydroxyl radicals than other MTs, which is likely due to its structural differences^28^.

The present study integrated single-cell RNA sequencing (scRNA-seq) and ATAC-seq data to reveal a distinct expression pattern of *Mt3* in the osteoclast population. The function of MT3 in osteoclast differentiation, bone resorption, and osteoporosis was investigated using *Mt3* knockout mice. To gain mechanistic insights, Metascape and Homer analyses of bulk RNA-seq of osteoclasts treated with *Mt3* siRNA were performed, which revealed ROS and the SP1 transcription factor as the molecular targets of MT3 in osteoclast regulation. The present results demonstrated that the MT3 zinc-binding protein is instrumental in regulating osteoclasts and plays a pivotal role in bone remodeling.

## Methods

### Mice

The Institutional Animal Care and Use Committee of Seoul National University approved all the animal experiments. *Mt3*^+/−^ mice on a C57BL/6 background, which were kindly provided by Prof. JY Koh (University of Ulsan, College of Medicine)^29^, were bred to produce both wild-type (*Mt3*^+/+^) and knockout (*Mt3*^−/−^) offspring. Mice were kept in the SPF animal facility with a 12-h light-dark cycle. In the ovariectomy (OVX)-induced osteoporosis model, 8-week-old female littermates were anesthetized and randomly assigned to receive either a sham operation (sham group) or bilateral ovariectomy (OVX group). After four weeks, the mice were sacrificed, and their femurs and L3 vertebrae were harvested for subsequent analyses.

### μCT analysis

Femurs and vertebral bones from *Mt3*^+/+^ and *Mt3*^–/–^ mice were analyzed by a SkyScan 1172 μCT scanner (Skyscan, Aartselaar, Belgium). By targeting a region of interest (ROI), three-dimensional images were created using CTVOX software (Bruker, USA). For the distal femurs, the trabecular bone properties were evaluated within a 1-mm-thick region located 0.7 mm beneath the growth plate. The assessed ROI bone parameters included BV/TV, Tb.Th, Tb.N, and Tb.Sp. A similar methodology was adopted for the analysis of vertebral bones.

### Histomorphometry

For histological analysis, femurs were fixed, decalcified, and embedded in paraffin. The hematoxylin and eosin (H&E) staining procedure was followed per standard practice. TRAP staining and methyl green counterstaining were performed as previously described^30^. The osteoclast number per bone perimeter (N.OC/B.Pm), osteoclast surface per bone surface (OC.S/B.S), and osteoblast number per bone perimeter (N.OB/B.Pm) were measured with Osteomeasure software (Osteometrics, USA).

### Osteoclast differentiation

BMMs were generated from bone marrow cells obtained from the tibiae and femurs of 5-week-old *Mt3*^+/+^ and *Mt3*^–/–^ mice as previously described^31^. BMMs were seeded at 2 × 10^4^ cells per well in 48-well plates or 2 × 10^5^ cells per well in 6-well plates in α-MEM supplemented with M-CSF (30 ng/mL). On the following day, the cells were treated with M-CSF and RANKL (100 ng/mL) for 5 days to stimulate osteoclast differentiation. TRAP staining was performed according to the manufacturer’s instructions (Sigma, 387A-1KT, USA). TRAP-positive cells containing more than three nuclei were considered mature osteoclasts. To investigate the effect of zinc on osteoclast differentiation, 1 μM TPEN with or without 1 μM ZnSO_4_ was added to the culture starting from Day 2. To examine the impact of zinc deficiency on SP1, cells were treated with 5 μM TPEN for 4 h on Day 3.

### Single-cell RNA-seq data analysis

Osteoclast culture scRNA-seq data (GSE147174) and mouse synovial mononuclear phagocyte scRNA-seq data (GSE134420) were obtained from the GEO database. Dimensionality reduction was performed using Seurat v.3. The scRNAtoolVis package was used to create volcano plots for highly variable genes across osteoclast clusters. The Monocle3 algorithm was used for pseudotemporal analysis, and SCENIC software was used for transcription factor analysis. Pearson correlation analysis was also performed.

### Bulk RNA-seq and data processing

Bulk RNA-seq was performed and analyzed under the guidance of E-Biogen Inc. (Seoul, Korea). Following siRNA treatment of the BMMs, the cells were cultured in osteoclast differentiation medium. After collecting the cells on Days 3 and 5, total RNA was extracted. The raw RNA-seq reads were aligned to the *Mus musculus* (mm10) reference genome. The DESeq2 package in R was used for analysis, with a *p* value < 0.05 and a fold change > ±1.2. The top 500 upregulated genes in the *Mt3* siRNA group were subjected to Metascape analysis. Homer analysis on the top 500 genes was performed using the findMotifs.pl script. This compilation of genes was provided to Homer in the form of a gene name file, with the mouse promoter set being the selected option. For the analysis of RNA-seq data from bone marrow failure patients (GSE152262), SP1-regulated genes were subjected to enrichment analysis with enrichKEGG.

### ATAC-seq and ChIP-seq data analyses

To assess chromatin accessibility, the ATAC-seq dataset (GSE211671) of RANKL-stimulated BMMs was analyzed. ATAC-seq and NRF2 ChIP-seq data (GSE188460) were uploaded to the Integrative Genomics Viewer (IGV). The targeting regions were set within 2000 base pairs (bps) of the transcription start sites (TSSs) for the metallothionein gene family (*Mt1, Mt2, Mt3*, and *Mt4*). In addition, the Homer algorithm was utilized to discern potential transcription factors that bind to accessible chromatin regions of *Mt3*. Potential binding sites were analyzed using the MEME Suite program.

### SP1 activity assay

SP1 activity in nuclear extracts was measured using an SP1 transcription factor ELISA kit (Active Motif, Japan), in which the primary antibody detects only active SP1 specifically bound to the consensus binding site oligonucleotides. After adding the HRP-conjugated secondary antibody and developing solution, a colorimetric readout at OD 450 nm was obtained.

### ROS measurement

For the detection of intracellular ROS, the H_2_DCFDA fluorescent probe was used. The cells were incubated with 10 µM H_2_DCFDA for 12 min and visualized using a confocal microscope (Olympus FV300, Tokyo, Japan).

### Measurements of intracellular Zn^2+^

Cells were treated with 2 μM FluoZin-3, a zinc-specific fluorescent probe (Thermo Fisher Scientific, USA), for 20 min according to the manufacturer’s instructions. The cells were then subjected to confocal microscopy or flow cytometry to compare Zn^2+^ levels. Flow cytometry data were collected and analyzed using FlowJo software (version v10.8.1).

### Statistical analysis

Quantitative results are presented as average values accompanied by the standard error of the mean (SEM). The significance of differences between two groups was evaluated using Student’s *t* test, and for analyses involving more than two groups, analysis of variance (ANOVA) was applied. A *p* value less than 0.05 was considered to indicate statistical significance.

Additional methods are described in the Supplementary Information. Supplementary Table 1 provides primer sequences used for real-time PCR and Supplementary Table 2 provides information on antibodies and reagents used in this study.

## Results

### MT3 is highly expressed in osteoclasts

**Fig. 1:**
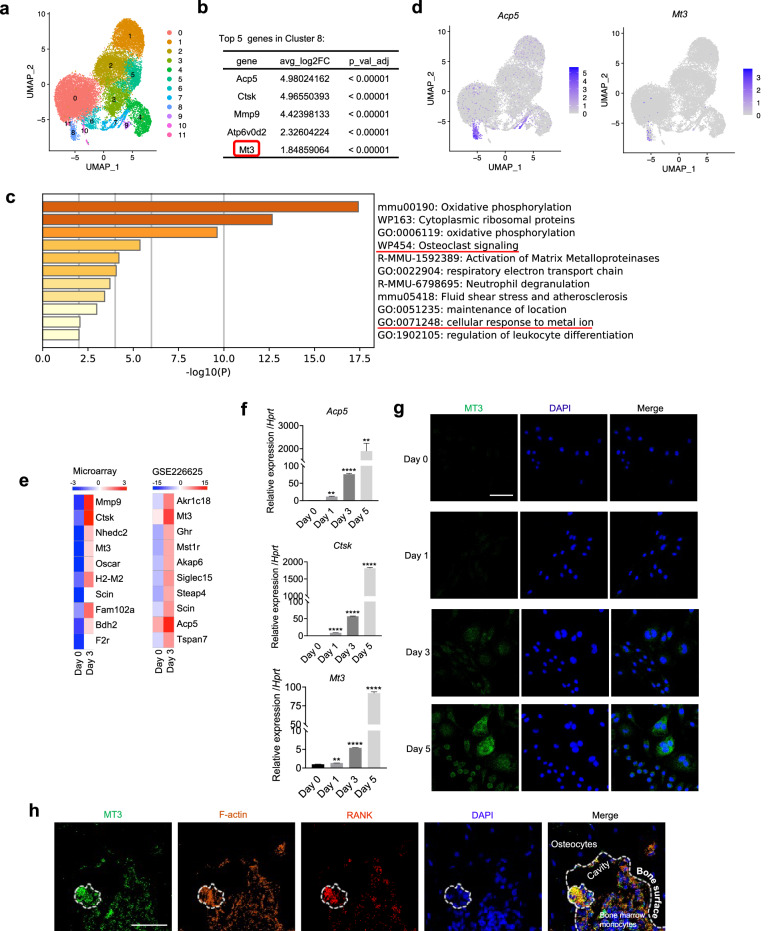
MT3 is upregulated during osteoclast differentiation. **a–d** Analyses of the scRNA-seq data of synovial mononuclear phagocytes (GSE134420). Uniform manifold approximation and projection (UMAP) visualization of 12 distinct clusters (**a**). Top 5 genes highly expressed in Cluster 8 (**b**). The results of the Metascape enrichment analysis are shown in ranked order for Cluster 8 (**c**). Gene expression overlaid on UMAP visualization (**d**). **e** Heatmaps depicting the top 10 genes with the highest fold changes in mRNA expression in RANKL-treated BMMs compared to untreated BMMs. (left, microarray data of our study; right, bulk RNA-seq data (GSE226625 dataset)). **f** Real-time PCR analysis of *Acp5*, *Ctsk*, and *Mt3* mRNA levels in BMMs cultured with RANKL for 1–5 days (*n* = 3). The data are shown as the mean ± SEM. *p < 0.05, **p < 0.01, ***p < 0.001, and ****p < 0.0001 according to a Student’s *t* test. **g** MT3 protein levels were assessed by immunofluorescence staining in BMMs cultured with RANKL. Scale bars, 50 μm. **h** MT3 expression in osteoclasts in femur sections shown by staining with MT3, F-actin, and RANK antibodies. Scale bars, 50 μm.

To elucidate novel regulators of bone metabolism, an unsupervised cluster analysis was performed on the scRNA-seq dataset (GSE134420)^32^ obtained from mononuclear phagocytic cells in the synovial tissue of serum-induced arthritis (SIA) model mice. The Seurat package was used to identify 12 distinct clusters (Fig. 1a). A cluster (Cluster 8) manifested characteristics of osteoclasts, as evidenced by enriched gene signatures associated with “osteoclast signaling” and pronounced overexpression of osteoclast marker genes, such as *Acp5, Ctsk*, and *Mmp9* (Fig. 1b–d; Supplementary Fig. 1a). Among the top five highly expressed genes in this cluster, *Mt3*—a gene previously unexplored in relation to osteoclasts—was identified (Fig. 1b). Notably, in addition to the “osteoclast signaling” term, enrichment of DEGs in the “cellular response to metal ion” category was identified in Cluster 8 (Fig. 1c). These findings suggested that metal ions may play significant roles in osteoclasts and that MT3, a critical intracellular metal-regulating protein, may constitute a vital signaling node for osteoclast differentiation and function.

The pronounced expression of *Mt3* in the osteoclast population in the SIA synovium agreed with our previous microarray analysis^31^ on BMMs compared to osteoclasts generated by RANKL treatment of BMMs. *Mt3* ranked fourth in the list of genes upregulated by RANKL (Fig. 1e, left), and this finding was further supported by the analysis of a recent public bulk RNA-seq database for osteoclasts^33^ (Fig. 1e, right). In this dataset, *Mt3* also exhibited a marked increase upon RANKL stimulation, ranking second among all upregulated genes (Fig. 1e). To validate these RNA sequencing data, real-time PCR was conducted and confirmed the increase in *Mt3* mRNA expression induced by RANKL in mouse BMM cultures (Fig. 1f). MT3-specific antibodies and immunofluorescence analyses verified that MT3 was localized in the nucleus and cytoplasm of cultured osteoclasts (Fig. 1g). A progressive increase in MT3 protein was evident on Days 3 and 5 of culture in RANKL-treated cells. This finding was further corroborated by high MT3 expression in osteoclasts in mouse femur sections (Fig. 1h). These results suggest a potential role of MT3 in osteoclasts.

### Osteoclast-specific MT3 expression is distinct from that of other MT family members

**Fig. 2:**
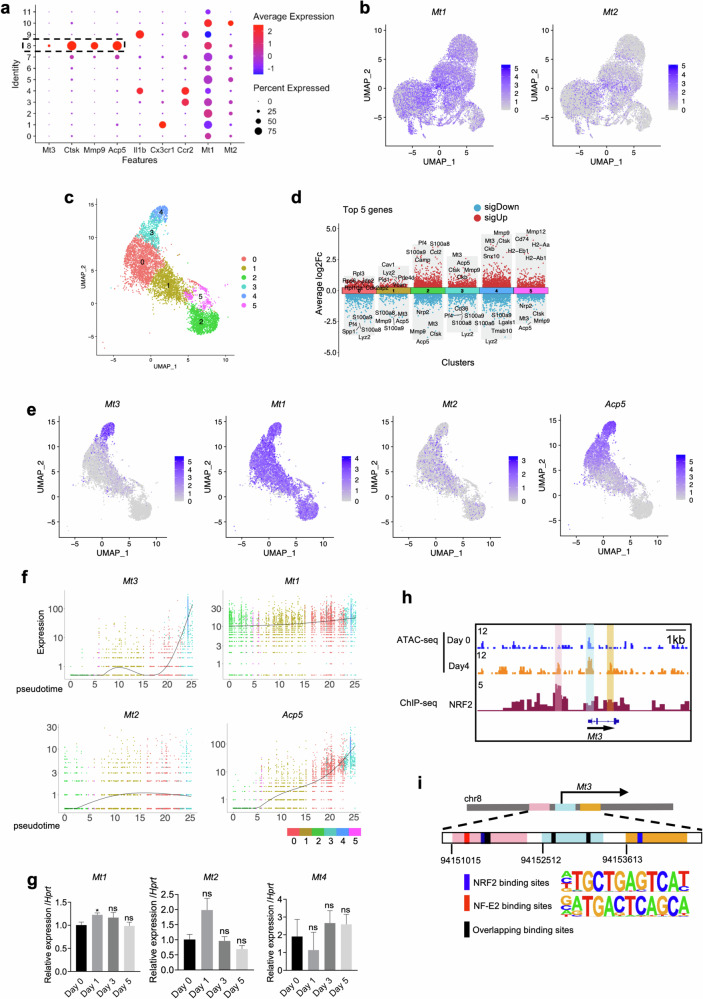
The expression pattern of MT3 during osteoclastogenesis is distinct from that of other MT family members. **a**, **b** Analyses of scRNA-seq data (GSE134420 dataset) derived from synovial mononuclear phagocytes. Dot plot representing the expression of selected genes in the clusters (**a**). The gene expression of *Mt1* and *Mt2* is presented by an UMAP (**b**). **c–f** Analyses of scRNA-seq data from differentiating osteoclast cultures (GSE147174). UMAP visualization of six clusters obtained after filtering the *Tnfrsf11a*^+^ *Csf1r*^+^ cells (**c**). Volcano plot of DEGs in clusters, and the top five DEGs in each cluster are labeled (**d**). Gene expression representation on UMAP (**e**). Dynamics of *Mt3, Mt1, Mt2*, and *Acp5* expression across the osteoclast differentiation trajectory based on pseudotime analysis (**f**). **g** Real-time PCR evaluation of *Mt1, Mt2*, and *Mt4* mRNA levels in BMMs following RANKL stimulation for 1–5 days (*n* = 3). **h** Genomic track visualization of ATAC-seq for osteoclasts on Day 0 and Day 4 (GSE211671 dataset), as well as NRF2 ChIP-seq profiles (GSE188460 dataset) near the TSS of the *Mt3* gene. Shaded areas highlight the accessible chromatin regions. **i** A schematic representation illustrates the potential transcription factor-binding motifs identified using Homer analysis and the predicted binding sites for NRF2 and NF-E2, as determined by MEME Suite analysis. All the data are shown as the mean ± SEM. *p < 0.05 according to Student’s *t* test; ns, not significant (**g**).

In further analyses of the scRNA-seq dataset of the SIA model, the expression patterns of MT family members were compared in different subsets of mononuclear phagocytes. In contrast to *Mt3* expression, which was restricted to the osteoclast cluster, *Mt1* and *Mt2* expression did not show specific patterns across various clusters, and *Mt4* was undetected (Fig. 2a, b).

To better understand the expression pattern of MTs during osteoclast differentiation, the present study evaluated the GSE147174 scRNA-seq data generated from differentiating osteoclasts obtained by culturing mouse BMMs with RANKL for 3 days^8^. Because *Tnfrsf11a* (encoding RANK) and *Csf1r* (encoding the M-CSF receptor) are essential for osteoclast differentiation, cells positive for both genes were extracted. Seurat analysis identified six clusters of these cells (Fig. 2c and Supplementary Fig. 1b). *Mt3* was prominently expressed in Clusters 3 and 4, which exhibited high expression of osteoclastic genes, such as *Acp5*, *Ctsk*, and *Mmp9* (Fig. 2d, e and Supplementary Fig. 1c, d). Pseudotime analysis using Monocle 3 predicted a differentiation path originating from Clusters 2 and 5, passing through Clusters 1 and 0, and reaching Clusters 3 and 4 (Fig. 2f and Supplementary Fig. 1e). This prediction was supported by Gene Ontology (GO) pathway enrichment analysis of the highly expressed genes in each cluster (Supplementary Fig. 1f) and agreed with the previous report^8^. Along the differentiation trajectory, *Mt3* exhibited an increasing expression pattern similar to that of *Acp5*, *Ctsk*, and *Mmp9* (Fig. 2f and Supplementary Fig. 1e). In contrast, the UMAP, violin plot, and pseudotime analyses of *Mt1* and *Mt2* did not reveal a unique expression pattern similar to that of *Mt3*, showing only minimal changes during osteoclast differentiation (Fig. 2e, f and Supplementary Fig. 1d). *Mt4* was not detected.

To validate the scRNA-seq-based results showing a differential pattern of MT expression, the mRNA level of each *Mt* was examined in BMMs treated with RANKL. *Mt1*, *Mt2*, and *Mt4* expression did not significantly change during osteoclast differentiation (Fig. 2g). Immunofluorescence experiments with an MT2 antibody showed no discernible difference across varying days of RANKL stimulation (Supplementary Fig. 2a). These comprehensive analyses revealed the unique upregulation pattern of MT3 during osteoclast differentiation, in contrast to the expression patterns of other MT family members.

To identify the transcription factors that may mediate MT3 induction by RANKL, the ATAC-seq data (GSE211671) generated from BMMs stimulated with RANKL were analyzed. The regions within 2000 bps from the TSSs of the MT family were evaluated to assess chromatin accessibility. There was an increase in chromatin accessibility in the *Mt3* gene upon RANKL stimulation, and this increase was not observed in the *Mt1* or *Mt2* gene (Fig. 2h and Supplementary Fig. 2b). Chromatin accessibility was not detected near the TSS of *Mt4*. The Homer algorithm^34^ was applied to the sequences of the accessible chromatin regions of *Mt3* to identify potential transcription factor-binding motifs. NRF2 and NF-E2 were among the three most likely binding motifs (Fig. 2i). These factors are regulators of various antioxidant genes, and they play vital roles in osteoclasts^35,36^. In addition, NRF2 ChIP-seq data analysis revealed that the open chromatin regions of *Mt3* served as binding sites for NRF2 (Fig. 2h). Further, the MEME analyses identified the potential binding sites for NRF2 and NF-E2 in the accessible chromatin regions (Fig. 2i). These findings suggested that NRF2 and NF-E2 may be intricately involved in the unique upregulation of *Mt3* by RANKL.

### MT3 has an inhibitory role in osteoclast differentiation and resorption

**Fig. 3:**
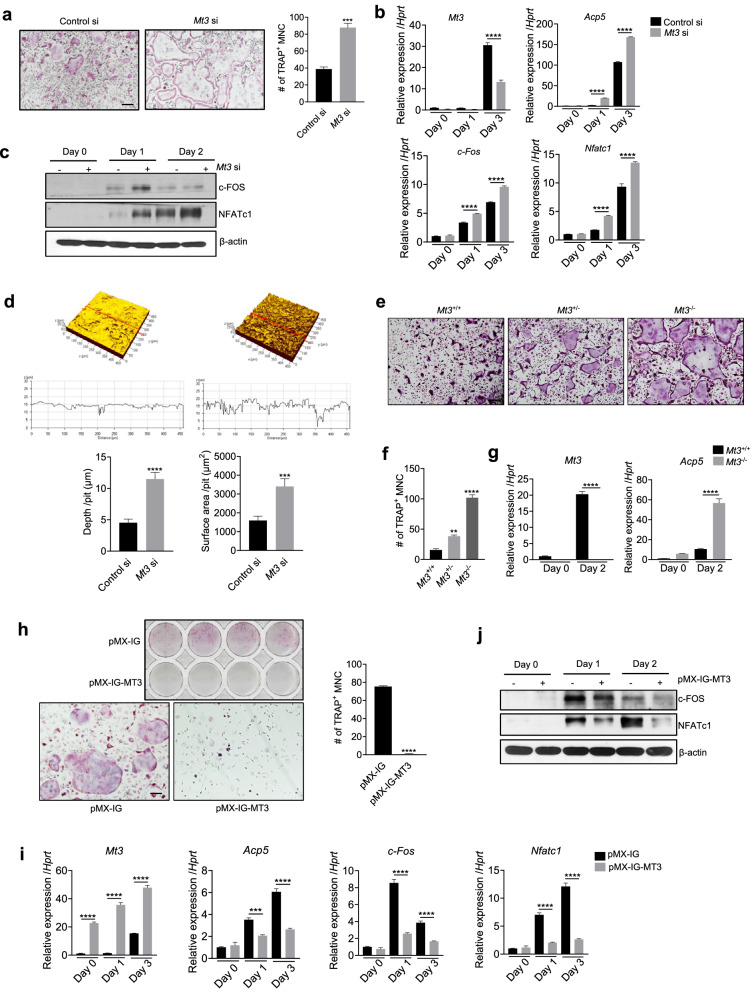
MT3 suppresses osteoclast differentiation and bone resorption. **a–d** BMMs transfected with control siRNA or *Mt3* siRNA were cultured in osteoclast differentiation medium. Representative TRAP-stained images (scale bars, 200 μm) and the numbers of TRAP-positive multinucleated cells with more than three nuclei (*n* = 4) (**a**). Real-time PCR analyses of the mRNA expression levels of *Mt3*, *Acp5*, c-*Fos*, and *Nfatc1* (*n* = 3) (**b**). Western blot analyses showing the protein levels of c-FOS and NFATc1 (**c**). Representative confocal microscopic scanning images of the dentin slices on which the transfected BMMs were cultured. The quantitated pit depth and resorption area are shown (*n* = 3) (**d**). **e–g** BMMs from *Mt3*^+/+^, *Mt3*^+/–^, and *Mt3*^–/–^ mice were cultured in osteoclast differentiation medium. Representative TRAP-stained osteoclasts. Scale bars, 200 μm (**e**). TRAP-positive multinucleated cells with more than three nuclei (*n* = 4) (**f**). Real-time PCR analyses of *Mt3* and *Acp5* mRNA (*n* = 3) (**g**). **h–j** BMMs were infected with retroviruses harboring pMX-IG-MT3 or the control vector, followed by treatment with RANKL. Top panel shows the TRAP staining of 48-well plates. Bottom panel shows the magnified images of representative TRAP staining. Scale bars, 200 μm. Right panel shows the quantification of TRAP-positive multinucleated cells (*n* = 4) (**h**). The expression levels of *Mt3*, *Acp5*, c-*Fos*, and *Nfatc1* mRNA were assessed by real-time PCR (*n* = 3) (**i**). The protein levels of c-FOS and NFATc1 were determined by Western blot analyses (**j**). All the data are shown as the mean ± SEM. *p < 0.05, **p < 0.01, ***p < 0.001, and ****p < 0.0001 according to Student’s *t* test (**a**, **d**, **h**) or one-way ANOVA with Bonferroni post hoc correction (**b**, **g**, **i**) or Dunnett’s test (**f**).

Given the upregulation of MT3 during osteoclastogenesis, the present study investigated the role of MT3 by evaluating osteoclast differentiation and function after manipulation of MT3 levels. The number of generated osteoclasts significantly increased (Fig. 3a) in *Mt3* knockdown cells (Fig. 3b and Supplementary Fig. 2c). Additionally, the expression of NFATc1 and c-Fos, which are pivotal transcription factors for osteoclast differentiation, was increased in *Mt3* knockdown cells at both the mRNA and protein levels (Fig. 3b, c). *Mt3* knockdown also significantly increased the resorption area and pit depth when the cells were cultured on dentine slices (Fig. 3d). The osteoclastogenic potential of BMMs derived from *Mt3*^+/+^, *Mt3*^+/–^, and *Mt3*^–/–^ mice was also evaluated. *Mt3* knockout markedly increased the number of TRAP^+^ mature osteoclasts (Fig. 3e, f) and increased the expression of *Acp5* (Fig. 3g). *Mt3*^+/–^ cells exhibited less osteoclast formation than *Mt3*^−/−^ cells but more than *Mt3*^+/+^ cells (Fig. 3f). The present study further investigated whether other MT family members possess similar osteoclast-inhibitory effects and found that neither *Mt1* nor *Mt2* knockdown promoted osteoclastogenesis (Supplementary Fig. 2d, e). In summary, *Mt3* knockdown or knockout promotes osteoclast differentiation and bone resorption.

To obtain further evidence for the function of MT3 as a negative modulator of osteoclastogenesis, the present study investigated whether MT3 overexpression suppresses osteoclast differentiation. A retroviral system was used to enhance MT3 expression in BMMs (Fig. 3i and Supplementary Fig. 2f), and the impact of MT3 overexpression on osteoclast differentiation was analyzed. MT3 overexpression markedly decreased the number of generated osteoclasts (Fig. 3h). Consistently, MT3 overexpression suppressed the expression of *Acp5*, *Nfatc1*, and *c-Fos* (Fig. 3i, j). Together, these findings confirmed the critical role of MT3 as an intrinsic negative regulator of osteoclastogenesis.

### *Mt3* knockout mice exhibit an osteopenic phenotype and intensified bone loss upon OVX

**Fig. 4:**
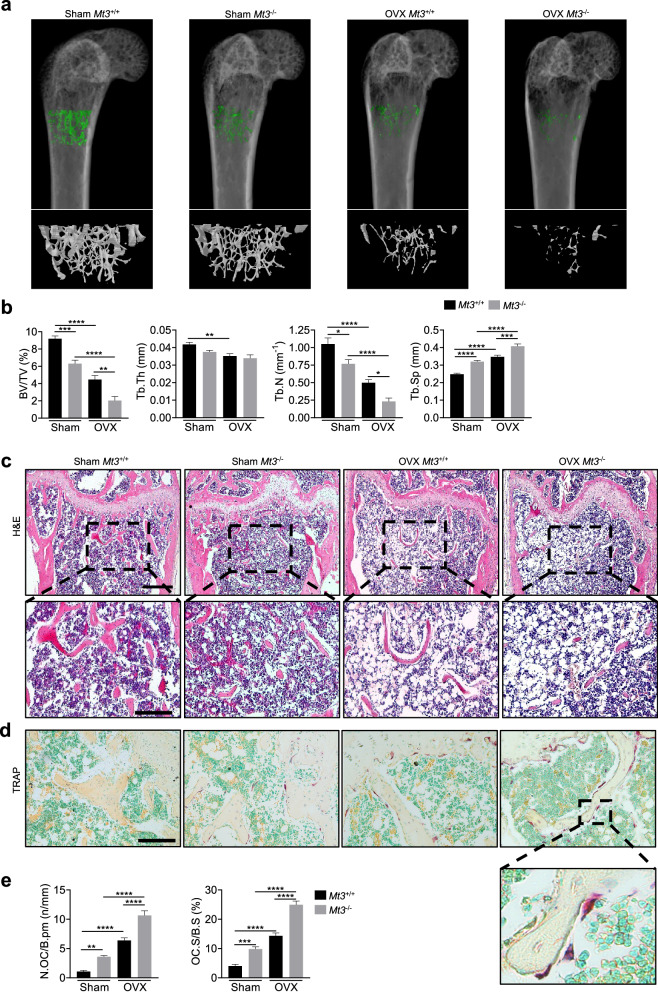
*Mt3* knockout exacerbates OVX-induced bone loss. **a** Representative 3D reconstruction images of µCT femoral bones from *Mt3*^+/+^ and *Mt3*^−/−^ sham-operated or ovariectomized mice. **b** Quantitative µCT analyses of various trabecular bone parameters, including BV/TV, Tb.N, Tb.Th, and Tb.Sp, in femoral metaphyses (*n* = 7–9). **c** H&E staining of the distal femur (top). Scale bars, 200 μm. Magnified region of interest (bottom). Scale bars, 100 μm. **d** Representative images showing TRAP-positive multinucleated osteoclasts. Scale bars, 100 μm. **e** Measurement of N.OC/B.Pm, and OC.S/B.S from TRAP-stained sections (*n* = 7–8). All the data are shown as the mean ± SEM. *p < 0.05, **p < 0.01, ***p < 0.001, and ****p < 0.0001 according to one-way ANOVA and Bonferroni post hoc correction (**b**, **e**).

Whether the function of MT3 is a critical determinant of bone metabolism under physiological and pathological conditions was investigated using *Mt3* knockout mice. Eight-week-old *Mt3*^*+/+*^ and *Mt3*^–/–^ mice were subjected to OVX or a sham operation. Four weeks after the operation, the femurs were analyzed by µCT. In the sham group, the trabecular bone volume per tissue volume (BV/TV), trabecular thickness (Tb.Th), and trabecular number (Tb.N) were lower but the trabecular separation (Tb.Sp) was greater in *Mt3*^–/–^ mice compared to *Mt3*^*+/+*^ mice (Fig. 4a, b), which indicated that bone mass was significantly reduced by *Mt3* gene deficiency. As expected, OVX induced bone loss in both *Mt3*^*+/+*^ and *Mt3*^–/–^ mice, while OVX-induced bone loss was further exacerbated in *Mt3*^–/–^ mice (Fig. 4a, b). µCT analyses of L3 vertebral bones confirmed the detrimental effect of *Mt3* deficiency on bone mass and quality (Supplementary Fig. 3a, b). Histological analyses of decalcified femur sections stained with H&E corroborated the µCT findings, showing a lower bone area in *Mt3*^−/−^ mice than in *Mt3*^+/+^ mice (Fig. 4c). To evaluate osteoclasts on the bone surface, decalcified sections were stained for TRAP activity (Fig. 4d). As expected, the number of TRAP-positive osteoclasts along the bone perimeter (N.OC/B.Pm) and osteoclast surface per bone surface (OC.S/B.S) increased in response to OVX (Fig. 4e). Further analysis revealed that these osteoclast parameters were greater in *Mt3*^–/–^ mice than in *Mt3*^*+/+*^ mice in both the sham-operated and OVX groups (Fig. 4e).

As *Mt3*^−/−^ mice lack MT3 expression not specifically in osteoclasts, we evaluated the potential osteoblast-mediated effects on the osteoporotic phenotype of the knockout mice. The number of osteoblasts per bone surface (N.OB/B.Pm) was not different between *Mt3*^*+/+*^ and *Mt3*^–/–^ mice (Supplementary Fig. 3c). No significant difference in the serum level of PINP, a biomarker of osteoblast activity, was observed between the two groups, while the serum level of CTX-1, a biomarker of osteoclast activity, was greater in *Mt3*^–/–^ mice compared to *Mt3*^*+/+*^ mice (Supplementary Fig. 3d). Furthermore, calcein double labeling revealed that bone anabolic activity was comparable between *Mt3*^*+/+*^ and *Mt3*^–/–^ mice (Supplementary Fig. 3e, f). These results suggested that the lower bone mass in the knockout mice is primarily due to *Mt3* deficiency in osteoclasts rather than in osteoblasts. Collectively, these findings indicated that MT3 plays a crucial role in preventing bone loss by limiting osteoclastogenesis in vivo, emphasizing its significance in bone metabolism.

### MT3 attenuates RANKL-induced ROS signaling

**Fig. 5:**
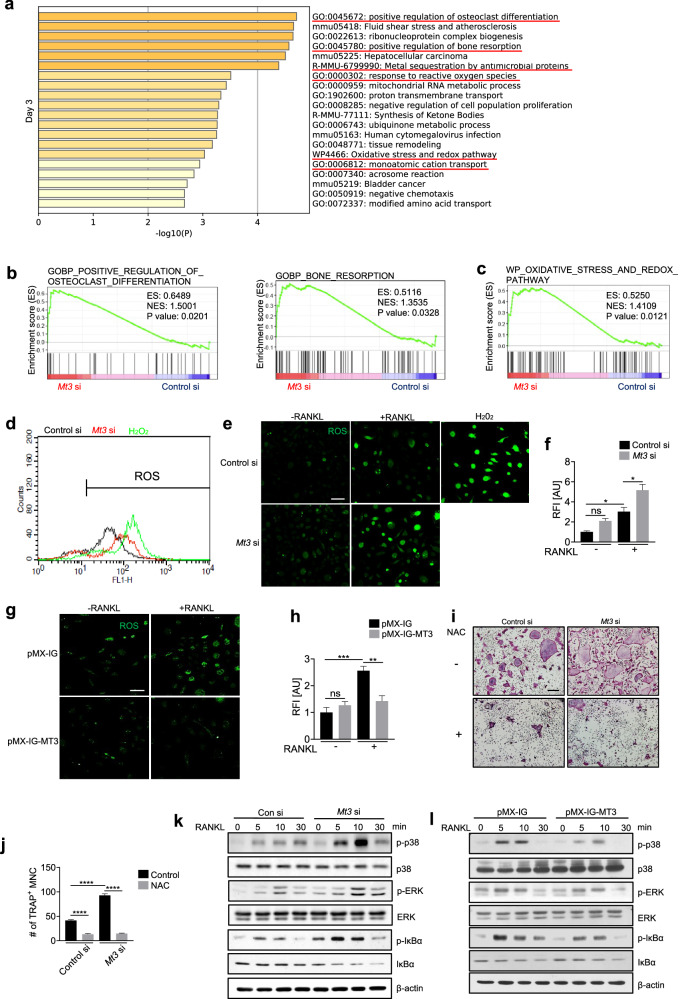
MT3-induced inhibition of osteoclast differentiation is mediated by a decrease in ROS. **a** Metascape enrichment analysis of the top 500 genes upregulated in *Mt3* knockdown cells treated with RANKL for 3 days. **b** GSEA of genes associated with “positive regulation of osteoclast differentiation” and “bone resorption” in cells after RANKL treatment for 5 days. **c** GSEA of “oxidative stress and redox pathways” in Day 5 samples. **d** Flow cytometry analysis of ROS levels in RANKL-treated cells in the control siRNA or *Mt3* siRNA group. **e**, **f** Representative immunofluorescence images and relative fluorescence intensity (RFI) of ROS following treatment with or without RANKL for 2 days (scale bars, 50 μm; *n* = 3). **g**, **h** Representative immunofluorescence images and RFI of ROS levels in cells in the pMX-IG or pMX-IG-MT3 groups after treatment with or without RANKL for 2 days (scale bars, 50 μm; *n* = 3). **i**, **j** Representative TRAP staining images and quantification of TRAP-positive multinucleated cells generated in the presence or absence of NAC (scale bars, 200 μm; *n* = 4). **k** Western blot analysis of BMMs transfected with either control or *Mt3* siRNA and treated with RANKL for the indicated period. **l** Western blot analysis of BMMs transduced with retroviruses harboring either pMX-IG or pMX-IG-MT3 and stimulated with RANKL for the indicated times. All the data are shown as the mean ± SEM. *p < 0.05, **p < 0.01, ***p < 0.001, and ****p < 0.0001 according to one-way ANOVA and Bonferroni post hoc correction (**f**, **h**, **j**).

To identify the potential mechanism that mediates the role of MT3 in osteoclasts, transcriptome analysis was performed to compare the expression profiles of osteoclasts with or without *Mt3* gene deficiency. BMMs transfected with control siRNA or *Mt3* siRNA were cultured in the presence of RANKL for 3 or 5 days. Based on a cutoff of a minimum 1.2-fold change and a *p* value less than 0.05, 726 upregulated genes on Day 3 and 1125 upregulated genes on Day 5 were identified in *Mt3* knockdown cells (Supplementary Fig. 4a). Key osteoclast marker genes were upregulated by *Mt3* knockdown, which was consistent with the inhibitory effects of MT3 on osteoclasts (Supplementary Fig. 4b, c). The top 500 genes with increased expression in *Mt3* siRNA group were selected for Metascape analyses^37^. The upregulated genes in the *Mt3* siRNA group were enriched in the “positive regulation of osteoclast differentiation” and “positive regulation of bone resorption” GO terms (Fig. 5a and Supplementary Fig. 4d). Additionally, associations between *Mt3* and the “oxidative stress and redox pathway” term as well as the “response to reactive oxygen species” term were identified (Fig. 5a). Gene set enrichment analysis (GSEA) indicated that similar GO biological pathways were more strongly enriched in the *Mt3* siRNA group than in the control siRNA group (Fig. 5b, c). Therefore, these findings indicated that MT3 deficiency leads to distinct biological alterations in oxidative stress and osteoclastogenesis.

Based on the sequencing-based pathway enrichment analysis and the antioxidant function of MT3 described in prior studies^25^, MT3 may influence osteoclasts via RANKL-induced ROS signaling. This potential mechanism was evaluated by determining ROS levels after *Mt3* knockdown. As previously reported^11,13^, RANKL stimulation significantly increased ROS generation (Fig. 5d–f and Supplementary Fig. 5a) and *Mt3* siRNA further enhanced the ROS signal (Fig. 5d–f), which was negated by N-acetyl cysteine (NAC), an ROS scavenger (Supplementary Fig. 5c). Consistent with this finding, the overexpression of MT3 decreased ROS levels (Fig. 5g, h and Supplementary Fig. 5b). In addition, NAC effectively inhibited the increase in TRAP-positive osteoclast numbers induced by *Mt3* siRNA (Fig. 5i, j). The MAPK and NF-κB pathways, which are essential for osteoclastogenesis and regulated by ROS^11,13^, were examined as potential downstream targets of the MT3-ROS axis in osteoclasts. RANKL-induced ERK and p38 phosphorylation was further enhanced by *Mt3* knockdown (Fig. 5k). *Mt3* knockdown also potentiated the effect of RANKL on the phosphorylation of IκB‐α and total IκB‐α levels (Fig. 5k). The opposite phenomena were observed with MT3 overexpression (Fig. 5l). Together, these results indicated that MT3 inhibits osteoclastogenesis, at least in part, by reducing the level of RANKL-induced ROS.

### MT3 represses SP1 activity in osteoclasts

**Fig. 6:**
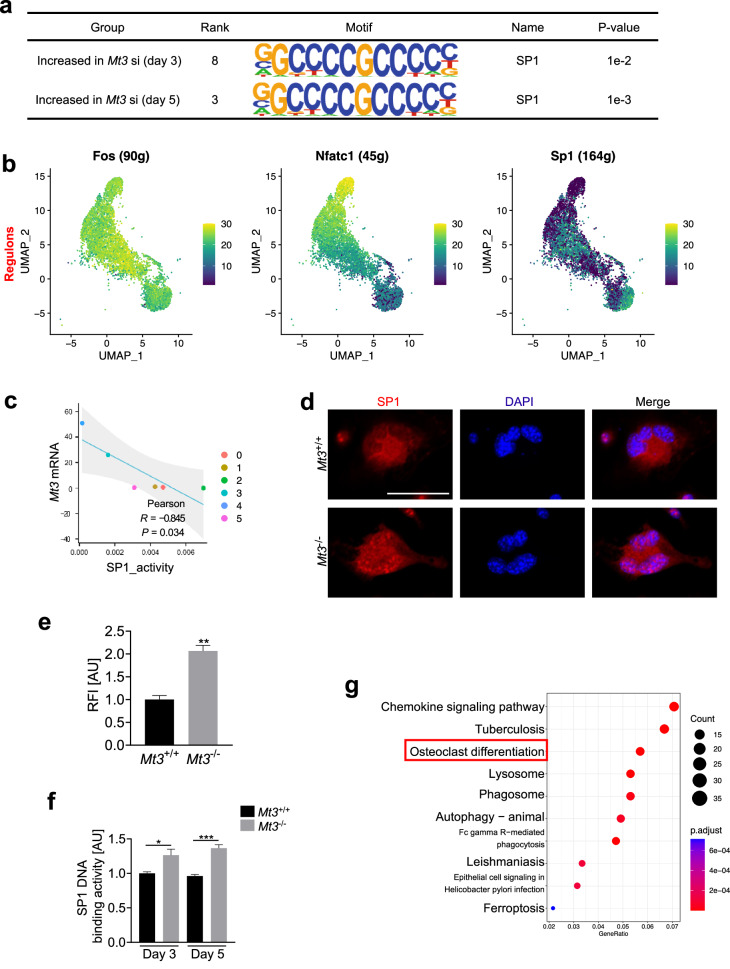
*Mt3* deficiency elevates SP1 transcription factor activity. **a** Homer analysis of transcription factor-binding site enrichment in the promoters of the top 500 genes upregulated in *Mt3* siRNA-transfected cells compared with control siRNA-transfected cells after osteoclastogenic culture. **b** Unsupervised graph-based clustering of SCENIC-derived gene regulatory network (GRN) scores for identified regulons onto the UMAP of the scRNAseq data of differentiating osteoclasts shown in Fig. 2c. **c** Pearson correlation coefficient and *p* value from linear regression analysis of *Mt3* mRNA levels and SP1 activity across the clusters of osteoclast cultures. **d** Immunofluorescence images of SP1 in osteoclasts on Day 5 of RANKL treatment. Scale bars, 20 μm. **e** Quantification of nuclear SP1 immunofluorescence signals (*n* = 3). **f** Analysis of SP1 DNA-binding activity in nuclear extracts from *Mt3*^+/+^ and *Mt3*^–/–^ cells after 3 or 5 days of RANKL treatment (*n* = 4). **g** Top 10 KEGG enrichment analysis pathways for 1071 upregulated genes associated with SP1 superactivation in white blood cells of patients with bone marrow failure. All the data are shown as the mean ± SEM. *p < 0.05, **p < 0.01, and ***p < 0.001 according to Student’s *t* test (**e**) or one-way ANOVA and Bonferroni post hoc correction (**f**).

Another established role of MT3 is the regulation of zinc homeostasis^25^. As various transcription factors bind zinc for their functions in diverse cell responses^17,18^, the regulation of transcription factors by MT3 in osteoclastogenesis was investigated by transcription factor-binding motif analysis using the Homer algorithm^34^ on genes upregulated in *Mt3* deficient cells. Among the top 10 enriched motifs, the transcriptional activity associated with the SP1-binding motif was upregulated (Fig. 6a and Supplementary Fig. 6a). SP1 proteins contain zinc finger domains near their carboxyl termini, which are involved in binding to high-GC content DNA sequences or GT/CACCC-box elements^38,39^.

To obtain an in-depth understanding of the activities of transcription factors involved in osteoclastogenesis, the SCENIC^40^ program was used to analyze the transcription factor regulation in single-cell clusters of differentiating osteoclast cultures (Fig. 2c). Consistent with previous findings, the transcriptional activity of c-Fos increased during the preosteoclast phase, followed by a slight decrease in the mature stage (Fig. 6b). The transcriptional activity of NFATc1 steadily increased, peaking in mature osteoclasts (Fig. 6b)^7,8^. Moreover, the transcriptional activity of SP1 decreased as differentiation progressed toward the mature phase (Fig. 6b). Among the clusters, SP1 activity was negatively correlated with *Mt3* expression (Fig. 6c). Among the other transcription factors predicted to mediate the upregulation of genes upon *Mt3* knockdown, the activity of NFATc1 was positively correlated with *Mt3* expression, and the activities of Myc and Max were negatively correlated with *Mt3* expression (Supplementary Fig. 6b). No significant relationships with *Mt3* were detected for other transcription factors (Supplementary Fig. 6b).

To evaluate whether MT3 affects the expression level of SP1 in osteoclasts, Western blot analysis was performed. There was a slight increase in total SP1 levels in *Mt3*^−/−^ cells compared with *Mt3*^+/+^ cells (Supplementary Fig. 6c). Western blot analysis of nuclear and cytoplasmic fractions revealed that the level of nuclear SP1 was higher in *Mt3*^−/−^ cells than in *Mt3*^+/+^ cells (Supplementary Fig. 6c, d), and immunofluorescence analyses further supported this result (Fig. 6d, e). In addition to evaluating the nuclear level of the SP1 protein, the DNA-binding activity, a critical element for its transcriptional function, of SP1 was evaluated^38,39^. The DNA-binding activity was enhanced in the nuclear extracts of *Mt3*-deficient osteoclasts (Fig. 6f). These computational and experimental results indicated that MT3 regulates the function of the SP1 transcription factor in osteoclast differentiation.

Given the inverse relationship between MT3 levels and SP1 activity during osteoclastogenesis, genome-wide sequencing data was searched to identify SP1 activity related to skeletal disorders. A recent study has reported a frameshift mutation in the *SP1* gene, which leads to highly elevated transcriptional activity of SP1 in patients with bone marrow failure^41^. RNA-seq data from blood cells of these patients (GSE152262 dataset), including 1071 upregulated genes in SP1-superactivated blood cells, were used to perform KEGG enrichment analysis. The analysis indicated that the osteoclast differentiation pathway was significantly overrepresented (Fig. 6g). Together, these results indicated that MT3 plays a repressive role in osteoclast differentiation by impacting SP1.

### MT3 affects SP1 activity through competitive Zn^2+^ binding for the regulation of osteoclastogenesis

SP1 features three Cys_2_His_2_ zinc fingers crucial for controlling gene expression via DNA binding^38,39^. Loss of Zn^2+^ due to its release or substitution with another metal ion can impair zinc fingers, as Zn^2+^ is essential for maintaining the structural and functional integrity of these domains^42^. RNA-seq data analysis identified enrichment of genes involved in pathways, such as “metal sequestration by antimicrobial proteins”, “monoatomic ion homeostasis”, and “regulation of monoatomic ion transport”, upon *Mt3* knockdown (Fig. 5a and Supplementary Fig. 4d). Given the high affinity of MT3 for metal ions, *Mt3* knockdown may disrupt the cellular balance and transport of monoatomic ions, including Zn^2+^. MTs play a crucial role in regulating intracellular Zn^2+^ availability by acting as Zn^2+^ stores to limit the amount of free Zn^2+^ and as Zn^2+^ distributors upon Zn^2+^ dissociation^20,43^. Thus, MT3 may modulate the function of SP1 by affecting intracellular Zn^2+^ levels.

**Fig. 7:**
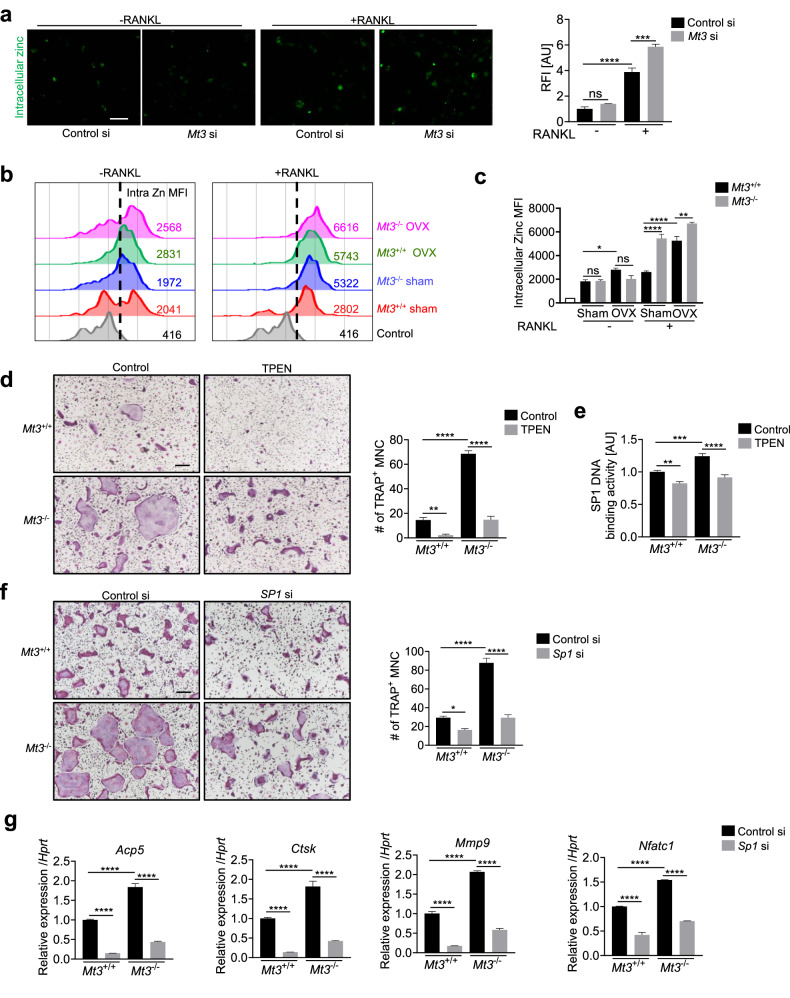
MT3 inhibits osteoclast differentiation by modulating SP1 activity via competitive Zn^2+^ binding. **a** The level of intracellular Zn^2+^ was assessed using the FluoZin-3 fluorescent probe (*n* = 3). Representative immunofluorescence images and fluorescence intensity of FluoZin-3-Zn^2+^ are shown. Scale bars, 50 μm. **b**, **c** Flow cytometric analysis of intracellular Zn^2+^ levels in BMMs from sham- or OVX-operated mice after culture with or without RANKL. The mean fluorescence intensity (MFI) of intracellular Zn^2+^ was determined via flow cytometry analyses (*n* = 3). **d** Representative TRAP staining images and quantification of TRAP-positive multinucleated cells in *Mt3*^+/+^ and *Mt3*^–/–^ osteoclast cultures with or without TPEN treatment (*n* = 4). Scale bars, 200 μm. **e** Analysis of SP1 DNA-binding activity in nuclear extracts from *Mt3*^+/+^ and *Mt3*^–/–^ osteoclasts with or without TPEN treatment (*n* = 4). **f** Representative TRAP staining images and quantification of TRAP-positive multinucleated cells in osteoclastogenic cultures of *Mt3*^+/+^ and *Mt3*^–/–^ BMMs transfected with control siRNA or *Sp1* siRNA (*n* = 4). Scale bars, 200 μm. **g** Real-time PCR analysis of *Acp5*, *Ctsk*, *Mmp9*, and *Nfatc1* mRNA levels (**f**) (*n* = 3). All the data are shown as the mean ± SEM. *p < 0.05, **p < 0.01, ***p < 0.001, and ****p < 0.0001 according to one-way ANOVA and Bonferroni post hoc correction (**a**, **c–g**).

To investigate whether cellular Zn^2+^ levels change during osteoclast differentiation, the FluoZin-3 fluorescence probe was employed. The level of intracellular free Zn^2+^ increased in response to RANKL treatment (Supplementary Fig. 6e), and Zn^2+^ levels were greater in *Mt3*^–/–^ osteoclasts than in *Mt3*^+/+^ osteoclasts (Supplementary Fig. 6f). *Mt3* knockdown osteoclasts also exhibited prominently elevated Zn^2+^ levels (Fig. 7a and Supplementary Fig. 6g). The Zn^2+^ levels in BMMs derived from sham and OVX *Mt3*^–/–^ and *Mt3*^+/+^ mice were measured by flow cytometry. Intracellular Zn^2+^ levels were greater in the OVX group than in the control group, which was further exacerbated by *Mt3* deficiency (Fig. 7b, c). These findings supported the critical role of MT3 in maintaining zinc homeostasis during osteoclast differentiation.

To investigate whether MT3-mediated modulation of Zn^2+^ levels affects the transcriptional function of SP1, the TPEN cell-permeable Zn^2+^ chelator was utilized. TPEN mitigated the enhanced osteoclast differentiation induced by *Mt3* knockout (Fig. 7d). Conversely, the addition of ZnSO_4_ partially reversed the inhibition of osteoclastogenesis by TPEN, highlighting the essential role of zinc in osteoclast differentiation (Supplementary Fig. 6h). Additionally, TPEN nullified the augmenting effect of *Mt3* knockout on SP1 DNA-binding activity (Fig. 7e). Similarly, *Sp1* siRNA transfection successfully reduced the increase in osteoclast differentiation induced by *Mt3* knockout (Fig. 7f and Supplementary Fig. 7a, b). Knockdown of the *Sp1* gene also attenuated the increase in MAPK activity in *Mt3*-deficient cells (Supplementary Fig. 7c). Subsequent examination of the mRNA levels of osteoclast marker genes confirmed that *Sp1* siRNA reversed the increase in osteoclastogenesis induced by *Mt3* knockout (Fig. 7g).

**Fig. 8:**
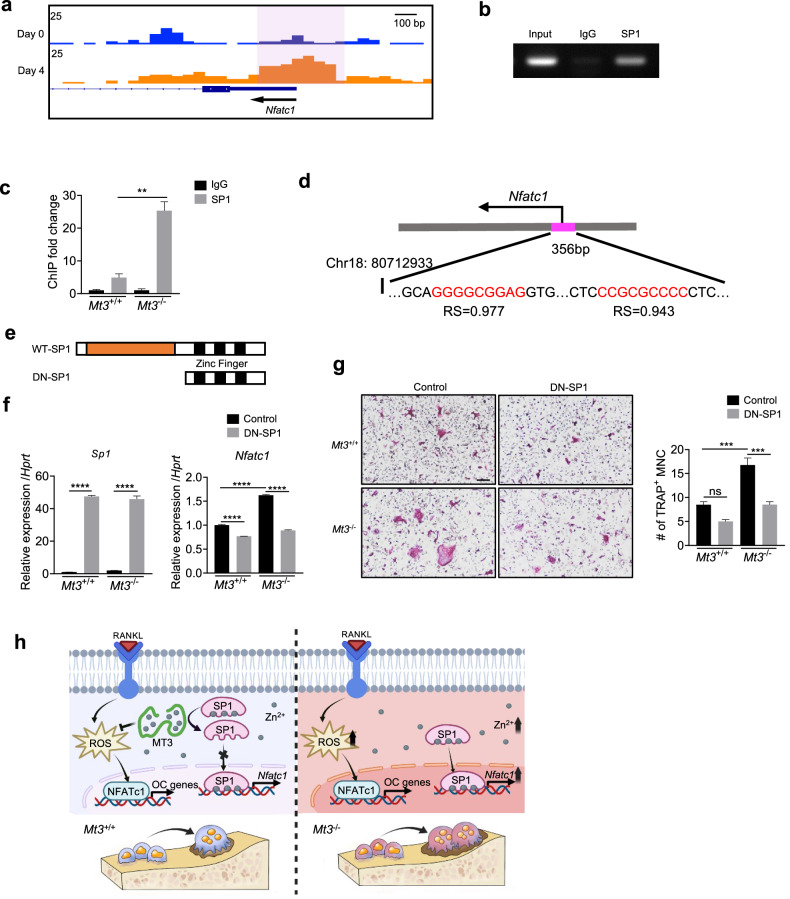
MT3 modulates osteoclast differentiation through SP1-mediated regulation of *Nfatc1.* **a** Genome track view of osteoclast ATAC-seq profiles (GSE211671) for *Nfatc1*. The region on Chr18:80712933-80713289 is highlighted with a pink background. **b** A representative image of ChIP-DNA electrophoresis. **c** ChIP‒qPCR results showing SP1 binding to the *Nfatc1* promoter (*n* = 3). **d** Analysis of the *Nfatc1* promoter region from (**a**) using JASPAR to identify potential SP1-binding sites. RS, relative score. **e** Schematic of DN-SP1. **f** Expression levels of *Sp1* and *Nfatc1* mRNA in BMMs infected with retroviruses carrying pMX-IG-DN-SP1 or pMX-IG followed by culture with RANKL (*n* = 3). **g** Representative stained images and quantification of TRAP-positive multinucleated cells (*n* = 4). **h** A schematic model depicting the role of MT3 in controlling excessive osteoclastogenesis.

To further explore the mechanism by which SP1 regulates osteoclasts, the potential role of SP1 for the gene expression of *Nfatc1*, a transcription factor crucial for osteoclastogenesis, was examined. Analysis of public ATAC-seq data (GSE211671 dataset) identified an increase in chromatin accessibility in the *Nfatc1* promoter region (chr18:80712933-80713289) after RANKL stimulation (Fig. 8a). To examine whether SP1 binds to the chromatin open region of *Nfatc1*, ChIP‒qPCR was performed with an SP1 antibody (Fig. 8b). The binding of SP1 to the *Nfatc1* promoter was markedly greater in *Mt3*^−/−^ osteoclasts than in WT osteoclasts (Fig. 8c). Consistently, two potential SP1-binding sites were identified in this region via JASPAR analyses (Fig. 8d). To further confirm the transcriptional regulation of *Nfatc1* by SP1, a dominant-negative inactive SP1 mutant (DN-SP1), which retains three zinc finger domains and competes for DNA binding but lacks transcription activation domains, was overexpressed (Fig. 8e, f). Overexpression of DN-SP1 negated the increasing effects of *Mt3* knockout on both *Nfatc1* levels and osteoclastogenesis (Fig. 8f, g). Taken together, these results indicated that the absence of MT3 enhances SP1 activity, which stimulates *Nfatc1* transcription, resulting in excessive osteoclastogenesis.

## Discussion

The present bioinformatics analysis of the scRNA-seq data revealed pronounced expression of *Mt3* in the osteoclast-specific population of synovial mononuclear phagocytic cells in the SIA model mice. In addition, pseudotime analysis of the scRNA-seq dataset of osteoclast culture revealed that the upregulated expression pattern of *Mt3* was similar to that of well-known osteoclast marker genes. In contrast, *Mt*1, *Mt*2, and *Mt*4 did not exhibit significant differences among synovial cell populations or during osteoclast differentiation. This distinction may be explained by the RANKL-specific increase in chromatin accessibility near the *Mt3* TSS, a phenomenon not observed with other MT family members. Further analysis of the chromatin region suggested that NRF2, known for its role in the oxidative stress response^35^, is likely a transcriptional regulator of *Mt3*. This finding provides an association between the antioxidative role of MT3 in osteoclasts and the regulatory functions of NRF2, which requires further exploration. As this distinct expression pattern may imply an exclusive role of MT3 in osteoclasts, the impact of gene knockdown on osteoclastogenesis was manifested only with *Mt3*, not with other *Mt* members. The unique role of MT3 in osteoclasts may not be solely attributed to its distinct expression pattern. MT3 is postulated to perform different roles from other isoforms in various cellular development processes and disease progression, potentially due to its unique structure, which is characterized by a greater number of sulfur groups and a distinct β-domain^25^. Recent discoveries have shown that apo-MT3 forms a more compact conformation than apo-MT1, which is facilitated by increased cysteine accessibility^44^, providing enhanced physiological stability on apo-MT3 and differential binding kinetics to metals and protein‒protein interactions^44^.

The uniqueness of MT3 among MT family members has gained attention in brain research, especially for Alzheimer’s disease, and MT3 is often referred to as the brain-specific MT^21,24,25^. In the adult rat brain, MT3 is expressed in neurons, astrocytes, oligodendrocytes, and microglia, and it is induced by LPS in oligodendrocytes and microglia^45–47^. In the developing mouse brain, MT3 is highly expressed in glia^48^ (https://cells.ucsc.edu/?ds=mouse-dev-brain&gene=). We previously discovered that brain-type creatine kinase (CK-B), known for its marked expression in the brain, is prominently expressed in osteoclasts and controls their functionality^49^. It will be important to determine whether this feature of high expression, shared by brain cells and osteoclasts, extends beyond MT3 and CK-B. Moreover, brain microglia and osteoclasts share the embryonic origin of erythromyeloid progenitor-derived macrophages^50,51^, and genetic variants in protein tyrosine kinase 2β are associated with both osteoporosis and Alzheimer’s disease, potentially by causing defects in actin reorganization in both osteoclasts and microglia^52^. These shared features between osteoclasts and brain cells may be intensified under pathological conditions caused by inflammation and aging.

In addition to the impact of *Mt3* deficiency on osteoclast differentiation and function in vitro, the present study demonstrated the in vivo significance of MT3-mediated regulation of osteoclasts by employing an OVX-induced osteoporosis model. *Mt3*^−/−^ mice displayed lower bone mass and more osteoclasts than *Mt3*^+/+^ mice. As the *Mt3*^−/−^ mice used in the present study were whole body knockouts, gene deficiency in cells other than osteoclasts may have contributed to the osteoporotic phenotype. However, the possibility of osteoblast-derived contributions was excluded by observing no difference in osteoblast differentiation or bone formation rates between *Mt3*^+/+^ and *Mt3*^−/−^ mice. The possibility that immune cells or fibroblasts influence the osteoporotic bone phenotype of *Mt3*^−/−^ mice cannot be ruled out. Although little evidence has been reported for any role of MT3 in these types of cells, a recent study has indicated a role of MT3 in IL4-induced macrophage polarization^53^. Further studies with cell type-specific *Mt3* knockout mice could clarify this issue. To obtain more in vivo relevance, the relationship between human hip bone mineral density (BMD) and MT3 expression levels in peripheral blood monocytes and progenitors of osteoclasts was evaluated using the GSE56814 data from 73 unrelated women^54^. Although the difference was not statistically significant (*p* = 0.0735), *MT3* mRNA levels tended to correlate with high BMD (Supplementary Fig. 8). This finding underscored the potential importance of MT3 in human bone health.

The inhibitory role of MT3 in osteoclastogenesis was elucidated through the modulation of two small molecules, namely, ROS and zinc, which have versatile effects on intracellular signaling and affect the functions of numerous proteins. The present transcriptomic analysis of RANKL-treated *Mt3*-deficient osteoclasts revealed elevated mRNA levels of genes associated with oxidative stress-related pathways. ROS act as key control nodes for osteoclast differentiation and bone resorption under physiological and pathological conditions^11,13,55^. MT3 is characterized by its high cysteine content, with up to 20 cysteine residues making up nearly 30% of its total amino acid content^25^. This high cysteine content endows MT3 with the ability to form deprotonated cysteine and disulfide bonds, which promote structural changes, significantly contributing to its ability to quench free radicals^22,23^. Furthermore, compared with other MT family members, MT3 has a distinct antioxidative effect, as it more strongly scavenges hydroxyl radicals^28^. ROS inhibition by protein tyrosine phosphatases has been suggested to be the molecular basis for the ROS-mediated activation of the MAPK and NF-κB pathways^56^. The present study demonstrated that *Mt3* deficiency led to elevated ROS levels and augmented activation of the MAPK and NF-κB pathways, while MT3 overexpression had the opposite effect. Thus, the antioxidant function of MT3 is one of the key mechanisms responsible for its inhibitory role in osteoclastogenesis.

A group of zinc-binding proteins belongs to a transcription factor family^19^. As *Mt3* deficiency elevated free Zn^2+^ in osteoclasts, the present study aimed to identify potential transcription factors regulated by MT3. Analysis using the Homer informatics program revealed that the SP1 binding site was significantly enriched in genes upregulated by *Mt3* knockdown. Subsequent bioinformatics and biochemical analyses supported the inverse relationship between MT3 expression and SP1 activity. Previous studies have demonstrated that purified MT proteins compete for Zn^2+^ bound to SP1 and inhibit the DNA-binding activity of SP1^57–59^. However, direct evidence substantiating this conclusion within a cellular context is lacking. The present data showing the regulation of intracellular Zn^2+^ levels and SP1 activity by MT3 modulation in osteoclasts supported the competitive relationship between MT3 and SP1 for Zn^2+^ binding, thereby implying an important role for zinc homeostasis in cell differentiation. In addition, the TPEN cell-permeable Zn^2+^ chelator reduced the increase in the DNA-binding activity of SP1 caused by *Mt3* knockout and suppressed osteoclastogenesis. In addition to DNA-binding activity, the nuclear translocation of SP1 was enhanced in *Mt3*-deficient osteoclasts. The zinc finger domains of SP1 have previously been shown to be required for its nuclear localization and to mediate its interaction with importin α^60–62^. As Zn^2+^ is vital to the proper structural organization of the zinc finger domain, MT3 regulation of Zn^2+^ affects both the nuclear localization and DNA binding of SP1.

The present study further demonstrated the significance of SP1 in the MT3-mediated suppression of osteoclastogenesis by showing that *Sp1* knockdown negated the increase in osteoclast differentiation induced by *Mt3* knockout. Moreover, transcriptome analysis indicated that the genes involved in osteoclast-related pathways were upregulated in blood cells from patients with mutations that cause high expression of SP1 target genes. However, the mechanism by which SP1 regulates osteoclasts has remained unclear. The present study identified two potential SP1-binding sites within the accessible chromatin region of the *Nfatc1* promoter. ChIP assays demonstrated that SP1 can bind to the *Nfatc1* promoter, and this activity was enhanced by *Mt3* deficiency. Consistently, the overexpression of the inactive SP1 mutant suppressed *Nfatc1* transcription and osteoclastogenesis. Therefore, the present study revealed a new mechanism for osteoclastogenesis, which is mediated by SP1 regulation of NFATc1. Together with the finding that an increase in MT3 is associated with a decrease in SP1 activity during osteoclast differentiation, the present results suggested a new theory that the MT3-Zn^2+^-SP1-NFATc1 axis constitutes an important mechanism for controlling excessive osteoclastogenesis.

In summary, the present study revealed the unique function of MT3 in osteoclasts and the intricate molecular mechanisms underlying its effect (Fig. 8h). The present findings demonstrated that MT3 serves as a suppressor of osteoclast differentiation and expanded the understanding of the complex interplay between metal homeostasis and transcriptional regulation in bone metabolism, thereby paving the way for innovative therapeutic strategies for bone diseases.

## Supplementary information

Supplementary Figures and Tables
