# Supplementary material for: Unique expression and critical role of metallothionein 3 in the control of osteoclastogenesis and osteoporosis
Source: Exp Mol Med. 2024 Aug 1;56(8):1791–806. doi: 10.1038/s12276-024-01290-3 (PMC11372110; doi:10.1038/s12276-024-01290-3)
Supplement: Supplementary file 1 — Supplementary Figures and Tables [file 12276_2024_1290_MOESM1_ESM.pdf]

Supplementary Information

Unique expression and critical role of metallothionein 3 in the control of osteoclastogenesis and osteoporosis

Shenzheng Mo *et al.*

Corresponding author: Hong-Hee Kim, hhbkim@snu.ac.kr

Supplementary Methods

Measurement of PINP and CTX-1

Blood was collected from mice and allowed to rest for 30 minutes before being centrifuged for 15 minutes at 4°C and 4000 rpm. The serum obtained was then used for analysis. Measurements were conducted using a PINP ELISA kit (AC-33F1, Immunodiagnostic Systems, USA) or a CTX-1 ELISA kit (AC-06F1, Immunodiagnostic Systems), following the manufacturer's instructions. A 4-parameter logistic curve fit was utilized to generate the mean calibration curve and calculate the concentrations of samples.

Calcein double labeling

8-week-old mice received intraperitoneal injections of 20 mg/kg calcein (Sigma-Aldrich), dissolved in 2% sodium bicarbonate, on the first and seventh days. Three days after the second injection, femurs were harvested and fixed in 4% paraformaldehyde, then embedded in methyl methacrylate (MMA). MMA resin blocks were sliced and subjected to confocal microscopy to visualize calcein deposited bands. Bone formation rate (BFR), mineral apposition rate (MAR), and mineralized surface per bone surface (MS/BS) were measured as previously described[1].

Bone resorption assay

BMMs were cultured on dentin slices (Immunodiagnostic Systems, Boldon, United Kingdom) in the presence of RANKL and M-CSF for nine days. Dentin slices were scanned using a Carl Zeiss LSM 5-PASCAL laser-scanning microscope, and the resultant images were analyzed using LSM 5 Image Browser software (Carl Zeiss Microimaging) for both the resorption area and pit depth.

Gene silencing and overexpression

For gene silencing, gene-specific siRNA oligonucleotides were procured from Invitrogen (Carlsbad, USA). BMMs were transfected with siRNA oligonucleotides (40 nM) using Lipofectamine RNAiMAX according to the provided protocol. *Sp1* siRNA was purchased from Thermo Fisher Scientific (Assay ID: s74196). The sequences of siRNA oligonucleotides were presented in pairs. *Mt1* siRNA, 5'-GUUCCACCCUGUUUACUAATT-3' and 5'-UUAGUAAACAGGGUGGAACTG-3'; *Mt2* siRNA, 5'-GCAAAGAGGCUUCCGACAATT-3'

and 5'-UUGUCGGAAGCCUCUUUGCAG-3'; *Mt3* siRNA, 5'-CCUACUGGUGGUUCCUGCACCUGCU-3' and 5'-AGCAGGUGCAGGAACCACCAGUAGG-3'. For overexpression, *Mt3* DNA was subcloned into the pMX-IG vector using BamHI/XhoI restriction sites. PCR amplification of *Mt3* cDNA was performed using the primers of which sequences are forward 5'-CGCGGATCCGCGGCCACCATGGACCCTGAGACCTGC-3' and reverse 5'-CGCTCGAGTCACTGGCAGCAGCTGCA-3' with template cDNA obtained by reverse transcription of osteoclast mRNA. For the creation of dominant negative SP1 (DN-SP1), which lacks the N-terminal activation domains, we designed DNA sequences based on the Consensus Coding Sequences (CCDS) of NCBI (CCDS37229.1). The synthesis of this DNA and subcloning into pMX-IG vector using EcoRI and XhoI sites were conducted by Bioneer (Daejeon, Korea). Retroviral packaging was achieved by transfecting Plat-E cells with the pMX vectors and culturing for two days. The culture medium with retroviruses was collected, passed through a 0.45-μm syringe filter (Sartorius, Göttingen, Germany), and applied to BMMs after mixing with 10 μg/ml hexadimethrine bromide (polybrene; Sigma, USA).

Reverse-transcription quantitative real-time PCR

Real-time PCR was performed as previously described[1]. The PCR cycle was consisted of a denaturation phase at 95°C for 3 seconds and an amplification phase at 60°C for 33 seconds. mRNA levels of genes were calculated using the $2^{-\Delta\Delta CT}$ method with normalization to *Hprt* levels. Supplemental Table 1 provides primer sequences used for real-time PCR analyses.

Chromatin immunoprecipitation (ChIP)

ChIP was performed with the ChIP kit (Cell Signaling, #9005) following the protocol provided by the manufacturer. Briefly, cells were treated with 37% formaldehyde to crosslink proteins to DNA. After incubation at room temperature for 10 minutes, glycine was added to stop the reaction. Following nuclear extraction, micrococcal nuclease was added to digest DNA and sonication was performed for 15 cycles of 30 seconds to achieve DNA fragments ranging from 150 to 900 bp. DNA fragmentation was verified by electrophoresis. For the input control, 2% of the sonicated sample was reserved. For chromatin immunoprecipitation, sonicated samples were incubated with SP1 antibody or control IgG at 4°C overnight. After adding ChIP-grade protein G magnetic beads, samples were

incubated 2 hours at 4°C with rotation. Precipitated beads were thoroughly washed and incubated in the ChIP elution buffer at 65°C for 45 minutes with gentle vortexing. Subsequently, 2 µl of Proteinase K was added, and the mixture was incubated at 65°C for 2 hours to digest proteins. The purified DNA was then subjected to DNA electrophoresis and qPCR.

Western blotting

Cell lysates were acquired by treating cells with RIPA lysis buffer. For the isolation of nuclear and cytoplasmic proteins, we used the NE-PER Nuclear and Cytoplasmic Extraction Kit (Thermo Fisher Scientific, USA), following the protocol furnished by the manufacturer. Equal amounts of proteins were subjected to western blotting as previously described[1].

Confocal microscopy

Cells were fixed with 3.7% formaldehyde, permeabilized with 0.1% Triton X-100, and subjected to a blocking step with 1% BSA in PBS. Subsequently, the cells were incubated at 4°C with the selected primary antibody. After rinsing with PBS, the cells were exposed to the secondary antibody for 2 hours, followed by counterstaining with 4′,6-diamidino-2-phenylindole (DAPI) to highlight nuclei. The prepared slides were examined under a Zeiss LSM 700 laser-scanning microscope.

[1] Kim, M. K. et al. Salt-inducible kinase 1 regulates bone anabolism via the CRTC1-CREB-Id1 axis. *Cell Death Dis*. **10**, 826 (2019).

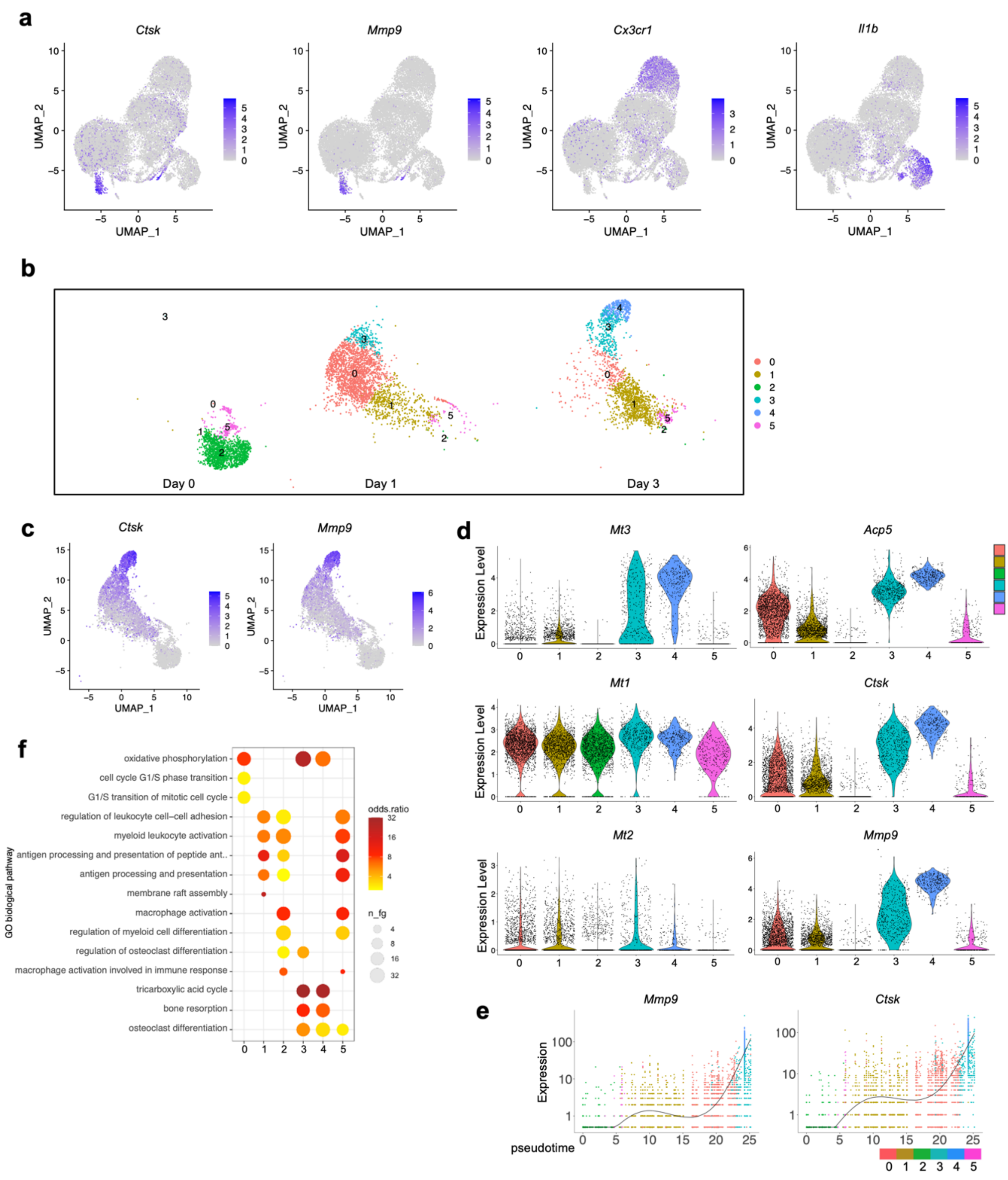

Supplementary Fig. 1 Analyses of scRNA-seq datasets: exploring gene expression in synovial mononuclear phagocytes and osteoclast subpopulations**.** **a** Analysis of scRNA-seq data (GSE134420) obtained with synovial mononuclear phagocytes of serum-induced arthritis (SIA) mice. Visualization of gene expression overlaid on UMAP. **b-f** Analysis of scRNA-seq data (GSE147174) of differentiating osteoclasts. UMAP visualization of data from day 0, day 1, and day 3 with RANKL (**b**). *Ctsk* and *Mmp9* expression in UMAP visualization of osteoclast subpopulations (**c**). Violin plots of gene expression across osteoclast subpopulations (**d**). *Mmp9* and *Ctsk* expression dynamics along the osteoclast differentiation trajectory (**e**). GO pathway enrichment analysis for highly expressed

genes within each osteoclast subpopulation, highlighting significant enrichments among cluster markers through color and circle size corresponding to the number of associated genes in each GO pathway (**f**).

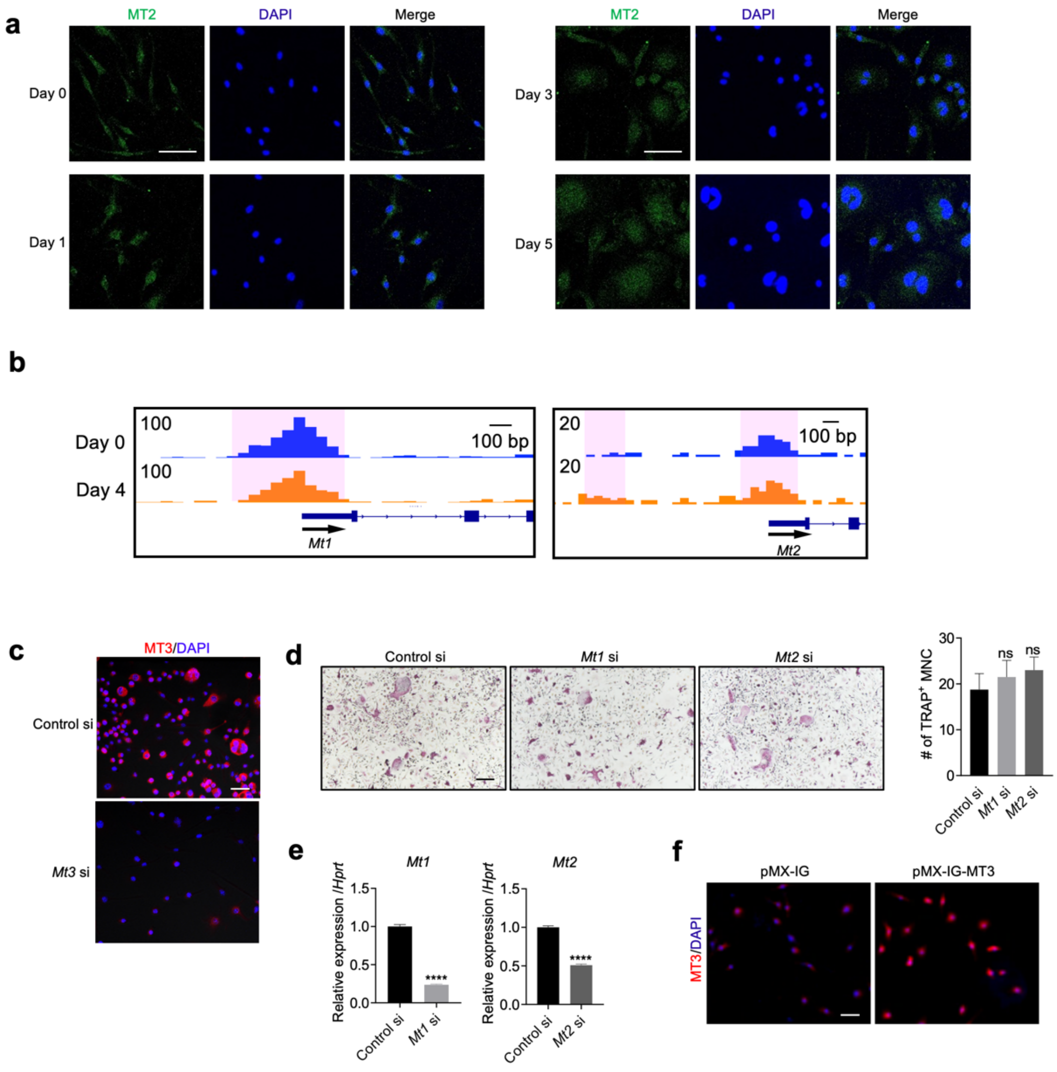

Supplementary Fig. 2 MT1 and MT2 have no effect on osteoclast differentiation. **a** Immunofluorescence assessment of MT2 expression in BMMs subjected to RANKL treatment for 5 days. Scale bars, 50μm. **b** Genomic track visualization of ATAC-seq analysis for osteoclasts on Day 0 and Day 4 (GSE211671). Day 0, BMMs at day 0 (unstimulated); Day 4, BMMs cultured with RANKL for 4 days. **c** Immunofluorescence analysis of MT3 expression in osteoclasts transfected with control siRNA or *Mt3* siRNA. Scare bars, 50 μm. **d** Representative TRAP staining images and quantification of TRAP-positive multinucleated osteoclasts generated from BMMs treated with control, *Mt1* and *Mt2* siRNA, n=4. Scare bars, 200 μm. Data are shown as mean ± SEM. ns, no significant difference; by one-way ANOVA and Dunnett's post hoc test. **e** Real-time PCR analyses of *Mt1* and *Mt2* mRNA in each knockdown cells, n=3. Data are shown as mean ± SEM. ****p < 0.0001; by Student's t test. **f** Immunofluorescence analysis of BMMs transduced with pMX-IG or pMX-IG-MT3. Scare bars, 50 μm.

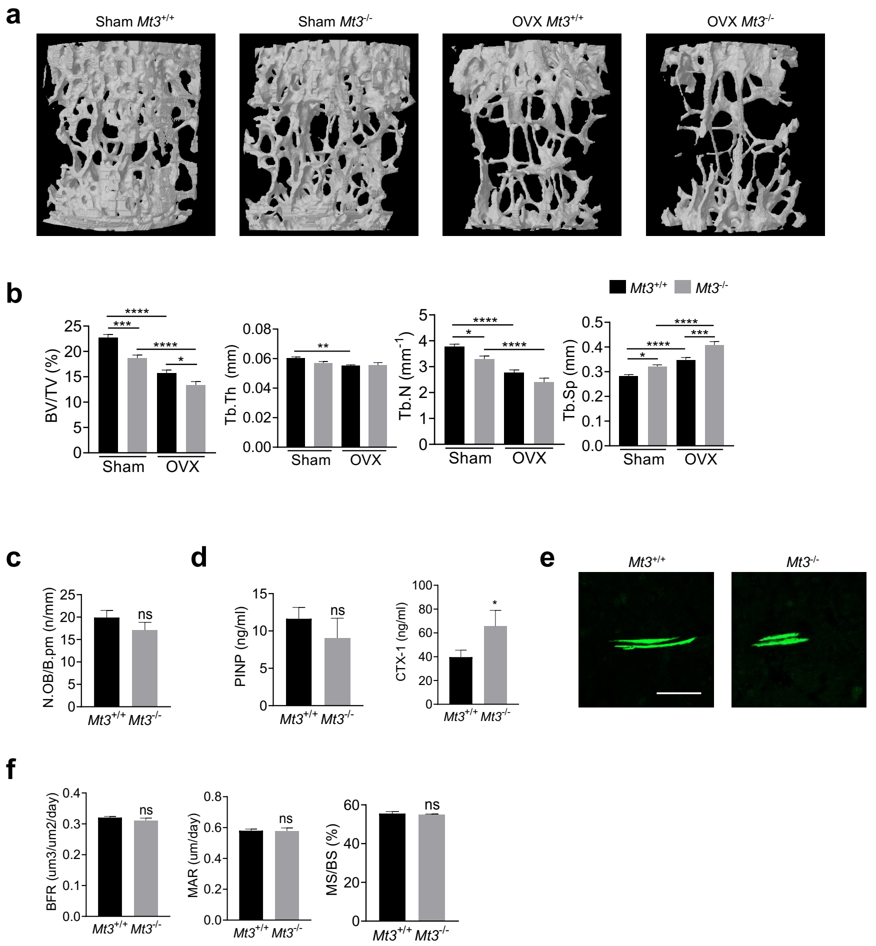

Supplementary Fig. 3 Vertebral bone from *Mt3* knockout mice display osteoporotic phenotype and osteoblast-related parameters are not different between *Mt3*$^{+/+}$ and *Mt3*$^{-/-}$ mice. **a** 3D images of vertebral bone for each group. **b** μCT analysis of diverse trabecular parameters. BV/TV, trabecular bone volume per tissue volume; Tb.Th, trabecular thickness; Tb.N, trabecular number; and Tb.Sp, trabecular separation, n=7-9. **c** Measurement of N.OB/B.Pm from H&E-stained sections, n=8. **d** ELISA measurement of PINP and CTX-1 levels in serum, n=3. **e** Representative fluorescence images of calcein double labeled femur sections. Scale bars, 100 μm. **f** Quantification of bone formation rate (BFR), mineral apposition rate (MAR), and mineralized surface per bone surface (MS/BS) parameters, n=3. All data are shown as mean ± SEM. *$p < 0.05$; **$p < 0.01$; ***$p < 0.001$; ****$p < 0.0001$; by one-way ANOVA and Bonferroni's post hoc test (**b**) or Student's t test (**c**, **d**, and **f**).

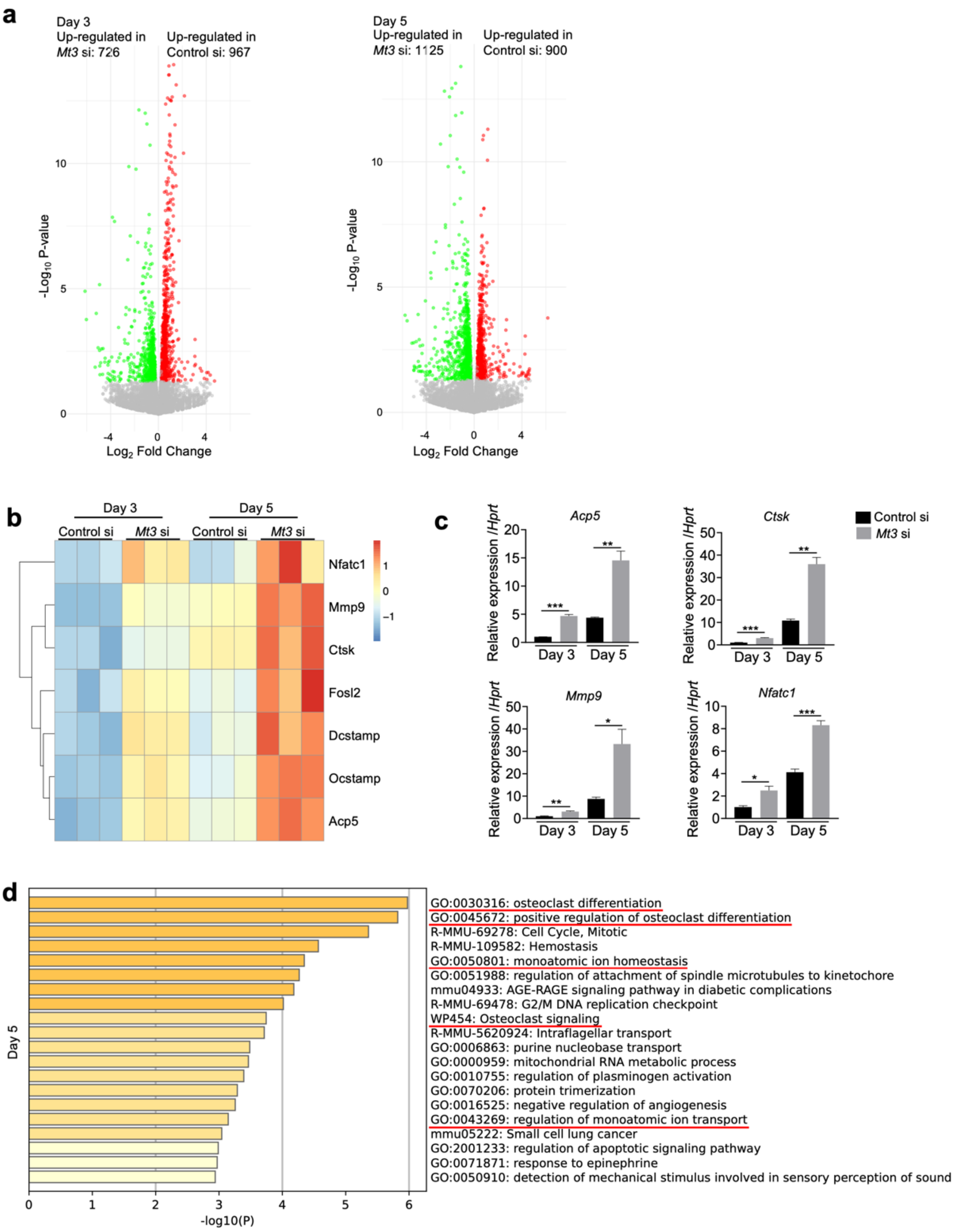

Supplementary Fig. 4 RNA-seq analyses comparing control and *Mt3* siRNA groups. **a** Volcano plots visualizing differences between control siRNA and *Mt3* siRNA groups. Genes upregulated in the control siRNA group (p-value < 0.05 and fold change > 1.2) are shown in red, whereas genes upregulated in the *Mt3* siRNA group are marked in green. **b** Heatmap showing osteoclast marker gene expression during osteoclast differentiation on day 3 and day 5. **c** Real-time PCR assessment of *Acp5*, *Ctsk*, *Mmp9*, and *Nfatc1* mRNA levels, n=3. **d** The top 500 upregulated genes in the *Mt3* siRNA group were subjected to enrichment analysis using Metascape with day 5 samples. The top 20

pathways are displayed, with key pathways underlined in red. Data are shown as mean ± SEM. *p < 0.05; **p < 0.01; ***p < 0.001; by Student's t test (**c**).

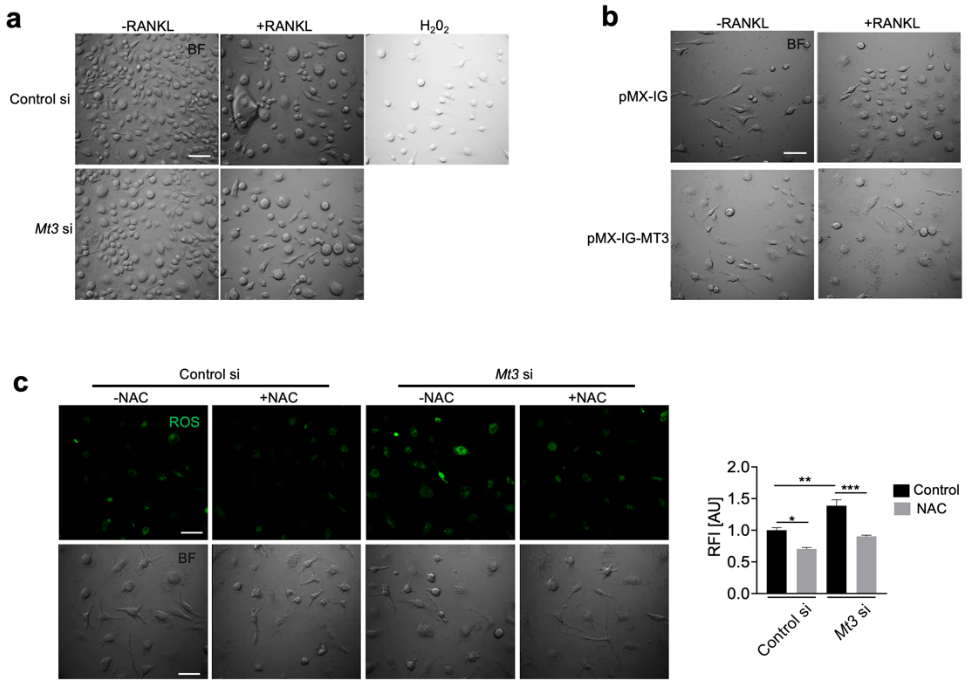

Supplementary Fig. 5 MT3 suppresses ROS in osteoclasts. **a** Bright-field images of Fig. 5e. Scale bars, 50 µm. **b** Bright-field images of Fig. 5g. Scale bars, 50 µm. **c** Representative immunofluorescence images of ROS levels in control siRNA and *Mt3* siRNA groups, with and without NAC treatment. Scale bars, 50 µm. The relative fluorescence intensity (RFI) of ROS is presented, n=3. All data are shown as mean ± SEM. *p < 0.05; **p < 0.01; ***p < 0.001; by one-way ANOVA and Bonferroni's post hoc test.

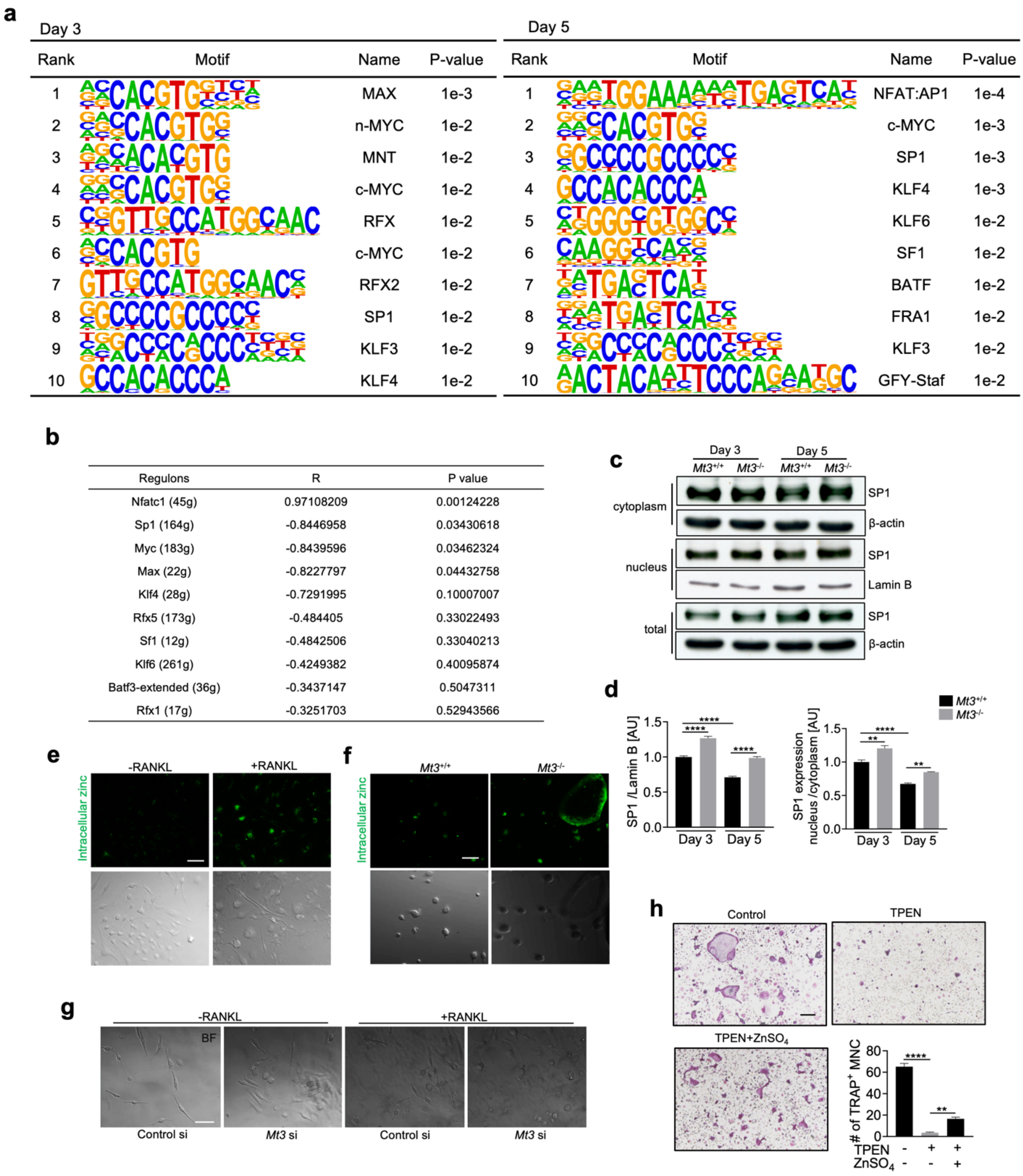

Supplementary Fig. 6 Regulation of SP1 by MT3 in osteoclasts. **a** Homer motif enrichment analysis for top 500 upregulated genes in *Mt3* siRNA cells on day 3 and day 5 after RANKL treatment. **b** Transcription factors from (**a**) that were characterized by SCENIC with inferred GRN scores. The linear relationship between their GRN scores and the average *Mt3* mRNA expression across each cluster was assessed using a Pearson correlation, followed by ranking the transcription factors based on their *p* values. **c** Western blot assessment of SP1 protein levels in the cytoplasmic, nuclear, and total cell lysates. **d** Relative intensity of SP1 Western blot bands (n=3). **e** Intracellular Zn^{2+} levels in BMMs with or without RANKL stimulation as measured with FluoZin-3. **f** Representative

immunofluorescence images of FluoZin-3-Zn^{2+} in osteoclasts from *Mt3*$^{+/+}$ and *Mt3*$^{-/-}$ cells. Scale bars, 50 μm. **g** Bright-field images of Fig. 7a. Scale bars, 50 μm. **h** Representative TRAP staining images and quantification of TRAP-positive multinucleated cells after treatment with TPEN together with or without $ZnSO_4$, n=4. Scale bars, 200 μm. Data are shown as mean ± SEM. **$p < 0.01$; ****$p < 0.0001$; by one-way ANOVA and Bonferroni's post hoc test (**d** and **h**).

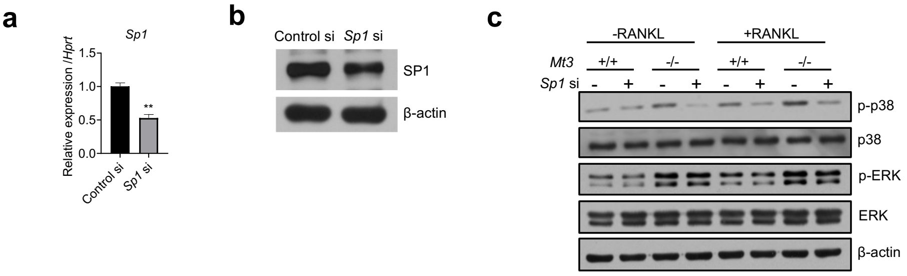

Supplementary Fig. 7 Involvement of SP1 in MT3 regulation of osteoclasts. **a** Expression of *Sp1* mRNA in osteoclasts transfected with control siRNA or Sp1 siRNA, n=3. Data are shown as mean ± SEM. **p < 0.01, by Student's t test. **b** Western blot assessment of SP1 protein levels. **c** Western blot analysis of osteoclasts transfected with control siRNA or Sp1 siRNA.

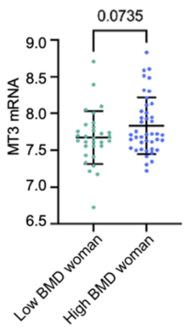

Supplementary Fig. 8 MT3 mRNA levels in circulating monocytes from individuals with low or high BMD (GSE56814). Data are shown as mean ± SEM. p value by Student's t test.

Supplementary Table. 1 Sequences of primers for real-time PCR

genes	Sequences (5'-3')	
Mt1	F	GGTCCTCTAAGCGTCACCAC
	R	GAGCAGTTGGGGTCCATTC
Mt2	F	CCAACTGCTCCTGTGCCT
	R	CTGGGAGCACTTCGCACA
Mt3	F	TACTGGTGGTTCCTGCACCT
	R	ATTTCTCGGCCTCTGCCT
Mt4	F	GCACAACCTGCAGCTGTAAA
	R	GCACACTTGGCACAGCCT
Acp5	F	CGACCATTGTTAGCCACATACG
	R	TCGTCCTGAAGATACTGCAGGTT
Ctsk	F	ATATGTGGGCCACCATGAAAGTT
	R	TCGTTCCCCACAGGAATCTCT
Mmp9	F	GACGGCACGCCTTGGTGTAG
	R	AGGAGCGGCCCTCAAAGATG
Nfatc1	F	CCAGTATACCAGCTCTGCCA
	R	GTGGGAAGTCAGAAGTGGGT
c-Fos	F	ACTTCTTGTTTCCGGC
	R	AGCTTCAGGGTAGGTG
Sp1	F	CTCCAGACCATTAACCTCAGTGC
	R	CACCACCAGATCCATGAAGACC
Hprt	F	CCTAAGATGAGCGCAAGTTGAA
	R	CCACAGGGACTAGAACACCTGCTAA
DN-*Sp1*	F	GGGAAGCGCTTTACACGTTC
	R	GGCCTCCCTTCTTGTTCTGG
Nfatc1	F	CCCCTCCCTTCAGTGACATC
(ChIP-qPCR)	R	CCTGTCCAAACCAGGGCG

Supplementary Table. 2 Information on antibodies and reagents

Antibody		
MT3	BIOSS	bs-4940R
c-FOS	Cell Signaling	2250S
RANK	Santa Cruz	sc-374360
NFATc1	Santa Cruz	sc-7294
β-actin	MBL International	M177-3
p-p38	Cell Signaling	9211S
p38	Cell Signaling	9212S
p-ERK	Cell Signaling	9101S
ERK	Cell Signaling	9102S
p-IκBα	Cell Signaling	2859S
IκBα	Cell Signaling	9242S
SP1	abcam	ab227383
Lamin B	Santa Cruz	sc-374015
Critical commercial assays		
H_2DCFDA	Thermo Fisher Scientific	C6827
FluoZin-3	Thermo Fisher Scientific	F24195
Nuclear and Cytoplasmic Extraction Reagents	Thermo Fisher Scientific	78833
Sp1 Transcription Factor ELISA Kits	ActiveMotif	41296
M-CSF	PeproTech	315-02
RANKL	PeproTech	315-11
